# Deciphering the roles of bacterial and fungal communities in the formation and quality of agarwood

**DOI:** 10.1007/s44154-024-00179-5

**Published:** 2024-09-20

**Authors:** Chen-Chen Fu, Bao-Xing Huang, Shan-Shan Wang, Yu-Chen Song, Dolkar Metok, Yu-Xiang Tan, Tai-Ping Fan, Alisdair R. Fernie, Meisam Zargar, Yan Wang, Mo-Xian Chen, Liang-Wen Yu, Fu-Yuan Zhu

**Affiliations:** 1 State Key Laboratory of Tree Genetics and Breeding, the Southern Modern Forestry Collaborative Innovation Center, Key Laboratory of State Forestry and Grassland Administration on Subtropical Forest Biodiversity Conservation, College of Life Sciences, Nanjing Forestry University, Nanjing, 210037 China; 2 Clinical Laboratory, Shenzhen Children’s Hospital, Shenzhen, 518000 China; 3 Dongguan Institute of Guangzhou University of Chinese Medicine, Dongguan Institute of Guangzhou University of Chinese Medicine, Dongguan, 523808 China; 4 Guangdong Province Key Laboratory for Biotechnology Drug Candidates, Institute of Basic Medical Sciences and Department of Biotechnology, School of Life Sciences and Biopharmaceutics, Guangdong Pharmaceutical University, Guangzhou, 510006 China; 5 CAS Key Laboratory of Quantitative Engineering Biology, Shenzhen Institute of Synthetic Biology, Shenzhen Institutes of Advanced Technology, Chinese Academy of Sciences, Shenzhen, 518055 China; 6 Department of Pharmacology, University of Cambridge, Cambridge, UK; 7 School of Health Sciences, Fuyao University of Science & Technology, Fuzhou, 350000 China; 8 Max Planck Institute of Molecular Plant Physiology, Am Mühlenberg 1, 14476 Potsdam-Golm, Germany; 9 Department of Agrobiotechnology, Institute of Agriculture, RUDN University, 117198 Moscow, Russia; 10 Science and Technology Innovation Center, Guangzhou University of Chinese Medicine, Guangzhou, Guangdong 510405 China; 11 State Key Laboratory of Desert and Oasis Ecology, Xinjiang Institute of Ecology and Geography, Chinese Academy of Sciences, Urumqi, 830011 China

**Keywords:** *Aquilaria sinensis*, Endophytic, Exophytic, Fungi, Bacteria, Plant tissue

## Abstract

*Aquilaria sinensis* is a significant resin-producing plant worldwide that is crucial for agarwood production. Agarwood has different qualities depending on the method with which it is formed, and the microbial community structures that are present during these methods are also diverse. Furthermore, the microbial communities of plants play crucial roles in determining their health and productivity. While previous studies have investigated the impact of microorganisms on agarwood formation, they lack comprehensiveness, particularly regarding the properties of the microbial community throughout the entire process from seedling to adult to incense formation. We collected roots, stems, leaves, flowers, fruits and other tissues from seedlings, healthy plants and agarwood-producing plants to address this gap and assess the dominant bacterial species in the microbial community structures of *A. sinensis* at different growth stages and their impacts on growth and agarwood formation. The bacteria and fungi in these tissues were classified and counted from different perspectives. The samples were sequenced using the Illumina sequencing platform, and sequence analyses and species annotations were performed using a range of bioinformatics tools to assess the plant community compositions. An additional comparison of the samples was conducted using diversity analyses to assess their differences. This research revealed that *Listeria*, *Kurtzmanomyces*, *Ascotaiwania*, *Acinetobacter*, *Sphingobium*, *Fonsecaea*, *Acrocalymma*, *Allorhizobium*, *Bacillus*, *Pseudomonas*, *Peethambara*, and *Debaryomyces* are potentially associated with the formation of agarwood. Overall, the data provided in this article help us understand the important roles played by bacteria and fungi in the growth and agarwood formation process of *A. sinensis*, will support the theoretical basis for the large-scale cultivation of *A. sinensis*, and provide a basis for further research on microbial community applications in agarwood production and beyond.

**Supplementary Information:**

The online version contains supplementary material available at 10.1007/s44154-024-00179-5.

## Introduction

Agarwood is a resinous, fragrant wood that is a basic component of some exclusive perfumes (Wang et al. 2019). Furthermore, agarwood is widely used as a medicine and incense in Asia, the Middle East, and Europe (Gao et al. 2023; Liao et al. 2018). The quality of agarwood can be judged by various methods, including its colour, fragrance, density, texture, and formation time (Ma et al. 2022). When agarwood appears brown or black, its content and grade are believed to be relatively high. *Aquilaria sinensis* is an evergreen tree that thrives in warm and dry climates; it is mainly distributed in three provinces of China, Hainan, Yunnan, and Guangdong, and it has high economic and medicinal value. Most *A. sinensis* trees must grow for more than 5 years to produce agarwood; however, the older the tree is, the longer the balsam takes to form and the better the quality of the balsam is (Chae et al. 2022; Liu et al. 2022a, b, c, d). Healthy *A. sinensis* trees cannot produce agarwood, as agarwood is only produced when the plant is damaged (Chen et al. 2018). Due to the high price and slow formation time of natural agarwood, many methods have been developed to artificially induce agarwood formation, including cutting, insect attack, drilling and chemical injection methods (Wang et al. 2022a, b). Among these methods, insect attacks on agarwood occur under long-term erosion and damage caused by insects, including microbial factors, which are important means of forming high-quality natural agarwood (Alamil et al. 2022).

Currently, three main hypotheses have been proposed regarding the formation of agarwood. The first is the "pathological" hypothesis, which suggests that agarwood formation is related to disease. Agarwood is believed to form as a result of fungal infection. The second hypothesis is the "trauma/pathological" hypothesis, which suggests that the primary cause of agarwood formation is physical trauma, with fungal infections being the secondary cause. The third hypothesis is the "nonpathological" hypothesis, which proposes that physical and chemical damage are the main causes of agarwood formation. This hypothesis suggests that both injuries and fungal invasions act as elicitors to induce defensive responses in *Aquilaria* trees. As a result of self-protection mechanisms, agarwood is formed through the generation of stress compounds. Simultaneously, the wounds become infected by microorganisms, and these microorganisms decompose the stress compounds into metabolites. The accumulation of these metabolites leads to the formation of agarwood (Subehan et al. 2005). Additionally, during the growth process of plants, the quantities of bacteria and fungi within them also increase, and the distributions of these bacteria and fungi vary among different tissues.

Plants contain different microbial communities in their bodies during different growth stages (Chen et al. 2021; Chen et al. 2017). These large microbial communities play important roles in stimulating host plant growth, antagonizing pathogens, tolerating stress, and controlling plant diseases (Liu et al. 2024; Tan et al. 2019). The association between the plant microbiome and resin formation is considered a result of plants adapting to natural microbial conditions. As a special plant, *A. sinensis* can secrete a special resin called agarwood(Zhang et al. 2020). Agarwood formation may be related to the defence mechanism of trees against physical damage, and physical damage can also lead to the invasion of microorganisms into trees (Liu et al. 2022a, b, c, d). The variety of microorganisms within plant bodies and those present in the surrounding environment on plant surfaces can influence the growth and development of plants (Liu et al. 2022a, b, c, d). Regarding *A. sinensis*, previous research has shown significant changes in the bacterial community structures after agarwood production, and agarwood formation is related to fungi that are associated with mould and decay (Duncan et al. 2017; Lee et al. 2019). Common fungi that infect agarwood include *Aspergillus*, *Botryosphaeria*, *Colletotrichum*, and *Penicillium*, as well as *Hypocreales*, *Fusarium*, and *Pleosporales* (Mohamed et al. 2014a, b). Although some research has been conducted on the bacteria and fungi present in *A. sinensis*, a complete introduction to the microbial community structures during the entire growth process of *A. sinensis* is currently unavailable (Yang et al. 2022; Zhang et al. 2014). Therefore, studying the microbial community compositions of *A. sinensis* during its growth and agarwood formation process is highly important. An analysis of publications related to *A. sinensis* in the past two years was conducted through searches of four major literature databases (e.g., PubMed, Web of Science, China National Knowledge Infrastructure, and the Wanfang Database). The number of publications on *A. sinensis* in recent years was significantly lower than that in previous years. Additionally, most studies have focused on the stem segments of *A. sinensis*, and a comprehensive exploration of the complete process from the plant's juvenile stage to adulthood and then to resin formation is currently unavailable. In this study, the Illumina sequencing platform was used to amplify the 16S rRNA gene and the internal transcribed spacer (ITS) region of *A. sinensis* tissues at different growth stages, with the goal of identifying the specific organisms present in the target plant (Zhang et al. 2020).

## Results

**Fig. 1:**
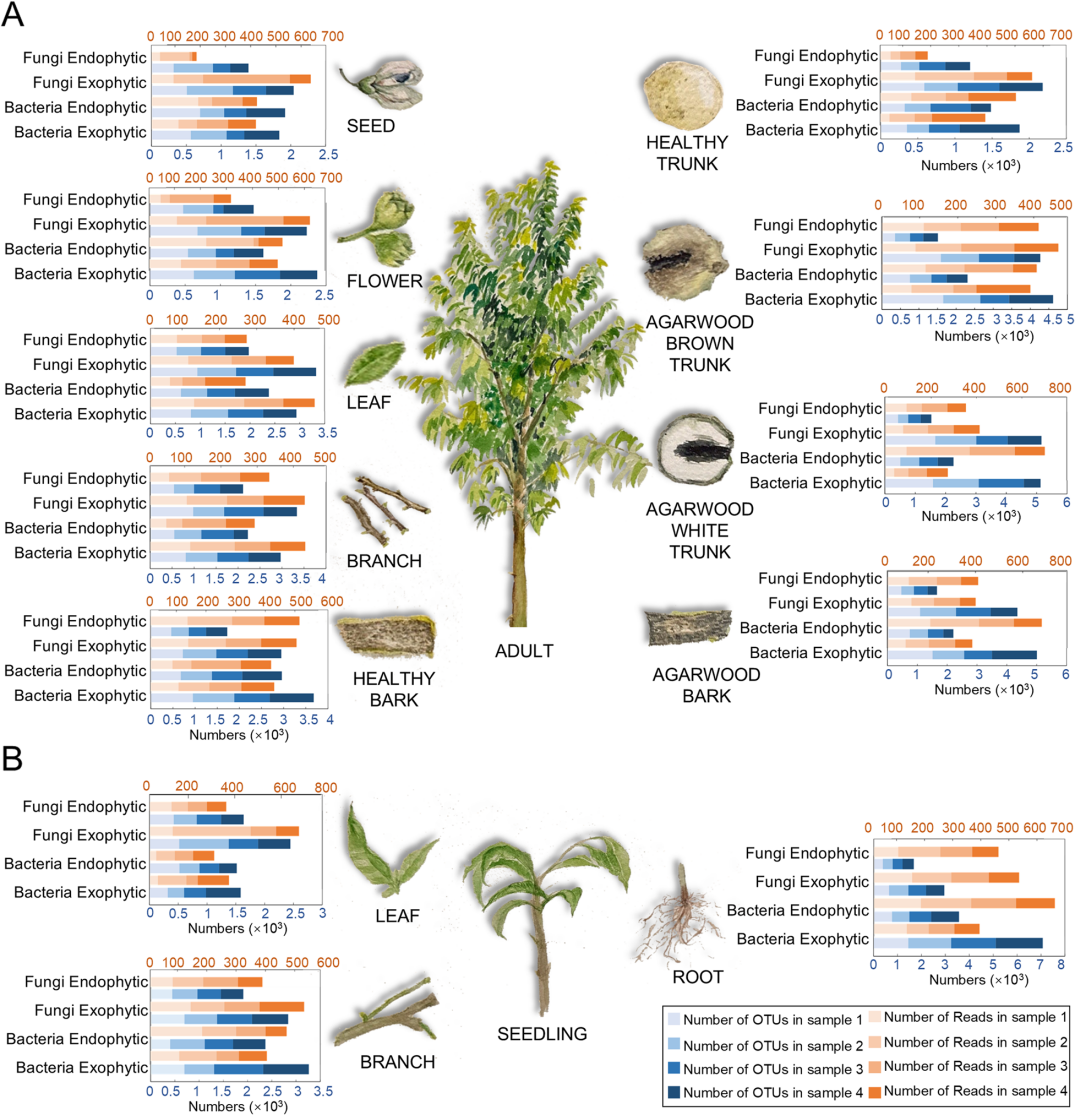
Differences in the number of OTUs and reads of bacteria/fungi in *A. sinensis* tissues. **A** Adult tissues of *A. sinensis* were collected, and each tissue sample had 4 replicates. We constructed bar charts of the numbers of OTUs and reads of the detected endophytic bacteria, exophytic bacteria, endophytic fungi and exophytic fungi to visually show the variations in the data for different tissues. The abscissa represents the numbers of OTUs and reads in various samples, and the ordinate represents the four microbial taxa. Red indicates the number of reads, and blue indicates the number of OTUs; from left to right, the four different shades of colour represent four different replicate samples. **B** Tissues of *A. sinensis* seedlings were collected, and each tissue had 4 replicates. We constructed bar charts of the numbers of OTUs and reads of the detected endophytic bacteria, exophytic bacteria, endophytic fungi and exophytic fungi to visually show the variations in data from different tissues. The abscissa represents the numbers of OTUs and reads in various samples, and the ordinate represents the four microbial taxa. Red indicates the number of reads, and blue indicates the number of OTUs; from left to right, the four different shades of colour represent four different replicate samples

The focus of this work was to determine why agarwood must grow to a certain age to produce a characteristic fragrance and to characterize the differences in the bacteria/fungi contained in the body of this tree during its development. We collected samples from different growth stages and different tissues of *A. sinensis* and analysed them to address this question (Fig. 1). Twelve groups of *A. sinensis* tissue were collected from Guangdong Province; for each tissue, four replicates were collected, which were divided into three groups according to different stages. The first group consisted of 5-month-old *A. sinensis* seedlings (young leaf/A1, young branch/A2, and young root/A3); the second group consisted of healthy 7-year-old *A. sinensis* (seed/B1, flower/B2, leaf/B3, branch/B4, bark/B5, and trunk/B6); and the third group consisted of mature *A. sinensis* exhibiting agarwood (white trunk/C1, brown trunk/C2, and agarwood bark/C3). After all the samples were collected, they were stored at -80°C prior to subsequent experiments.

### Cluster analysis of the microbiome operational taxonomic unit (OTU) number and community abundances in different tissues

After collecting the samples as described above, we conducted a comprehensive study of *A. sinensis* using community composition analyses, species abundance clustering analyses, and diversity analyses of different growth stages (e.g., seedlings, healthy adults, and agarwood) and different tissues (e.g., leaves and trunks, trunks and bark). First, we counted the number of reads and OTUs in 12 groups of samples (Table 1). In this table, plant tissues exposed to air have more exogenous fungi than those that are enclosed internally. Overall, bacteria outnumbered fungi in all tissues. Additionally, the differences in the microbial community OTUs and reads among the four replicate samples of each of the 12 tissues of *A. sinensis* were visually displayed using bar charts (Fig. 1; Table 1).

**Table 1:** The average reads and the number of OTUs in each tissue of *A. sinensis*

Sample ID	Endophytic Bacteria		Exophytic Bacteria		Endophytic Fungi		Exophytic Fungi	
	No. of OTUs	No. of seqs	No. of OTUs	No. of seqs	No. of OTUs	No. of seqs	No. of OTUs	No. of seqs
A1	882	299,867	1389	511,802	738	325,902	1214	639,615
A2	1297	481,557	1588	471,615	910	276,683	1636	411,621
A3	1880	663,381	1459	440,625	929	338,506	1608	439,037
B1	1190	424,038	1115	395,744	595	178,849	1189	571,542
B2	934	530886	1654	418,715	786	503,797	1432	493,955
B3	1237	273,075	1068	419,917	739	181,579	1163	639,553
B4	1228	296,839	923	368,372	765	355,602	1424	692,321
B5	1408	407,996	1661	411,898	854	396,127	1379	543,054
B6	842	510,760	3138	387,637	777	456,877	1427	532,454
C1	1243	417,435	2746	400,234	716	422,639	2150	475,692
C2	1163	703,848	2711	277,277	727	356,608	2451	417,058
C3	1195	687,898	2366	378,500	792	404,416	2016	393,389

**Fig. 2:**
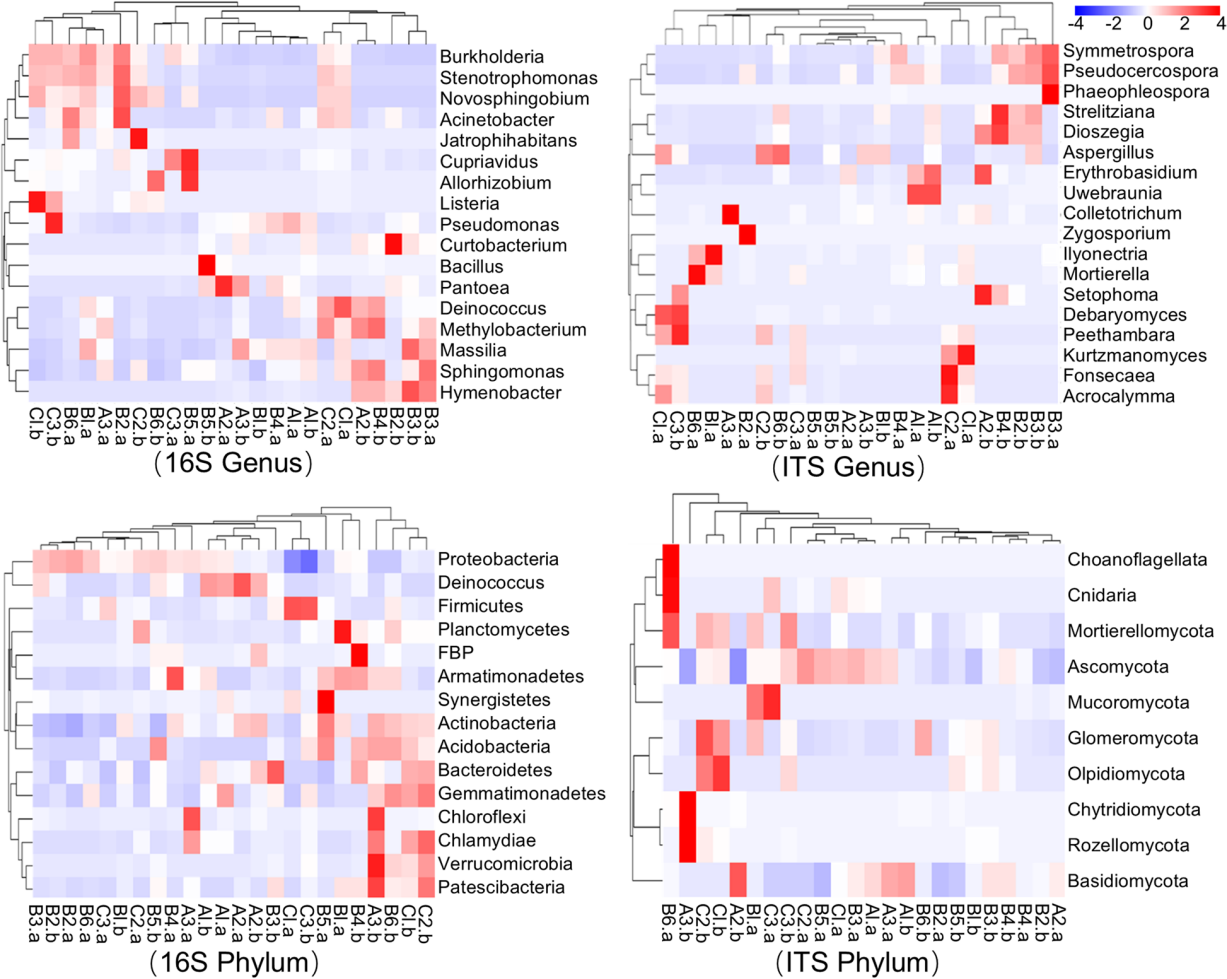
Clustering analysis of the microbial species abundances at the genus and phylum levels in various *A. sinensis* tissues. The horizontal axis represents the samples, and the vertical axis represents the species. The clustering tree on the left is the species clustering tree, and the clustering tree on the top reflects the similarity of the community composition between samples. The values corresponding to the middle squares are the standardized relative abundance of each row of species. The colour intensity of the squares represents the species abundances: the redder the square is, the greater the relative abundance of the species among the samples; the bluer the square is, the lower the relative abundance of the species among the samples. A horizontal comparison can be performed, but a vertical comparison cannot. The first group consisted of 5-month-old *A. sinensis* seedlings (young leaf/A1, young branch/A2, young root/A3); the second group consisted of healthy 7-year-old *A. sinensis* (seed/B1, flower/B2, leaf/B3, branch/B4, bark/B5, trunk/B6); and the third group consisted of mature *A. sinensis* with agarwood (white trunk/C1, brown trunk/C2, agarwood bark/C3). “a” indicates an endophyte, and “b” indicates an exophyte

A species abundance clustering analysis (Fig. 2) was conducted to understand the community composition and abundance information for various organizations. A correlation analysis of the microbiota and plant tissues revealed correlations among the top 20 bacterial and fungal genera/phyla and various tissues. The species abundance clustering heatmaps for the taxa at the kingdom, class, order, and family levels are provided in Supplementary Figure S3. An assessment of the endophytic bacteria at the genus level revealed that young leaves, young branches, and branches clustered together, seeds and leaves clustered together, flowers and brown trunks clustered together, and white trunks and agarwood bark clustered together. Young leaves and young branches were both obtained from *A. sinensis* seedling tissues; young branches and branches were both obtained from branches; seeds and leaves were obtained from healthy *A. sinensis* seeds and leaves, respectively; and white trunks and agarwood bark were obtained from agarwood *A. sinensis* tissues. The analysis of the species clustering tree revealed that *Listeria* and *Pseudomonas* clustered together, and these two genera were most abundant in the white trunk and agarwood bark samples, respectively. *Chryseobacterium*, *Sphingobium*, *Allorhizobium*, *Cupriavidus*, and *Dyella* also clustered together, with relatively high abundances in young roots, flowers, and brown trunks. Similarly, *Massilia*, *Sphingomonas*, *Methylobacterium*, and *Deinococcus* clustered together but were more abundant in the young leaves, young branches, and branches.

Assessments of the endophytic bacteria at the phylum level revealed that young leaves and young branches clustered together; flowers, leaves, and trunks clustered together; and brown trunks and agarwood bark clustered together. These three groups are derived from seedlings, healthy plants, and agarwood *A. sinensis* plants, respectively, and thus their similarities are highly consistent with their biological circumstances. Chloroflexi and Chlamydiae were most abundant in the seedling roots. Patescibacteria, Planctomycetes, and Verrucomicrobia clustered together, with the highest contents in seeds. However, Actinobacteria, Acidobacteria, and Synergistetes clustered together with higher contents in healthy *A. sinensis* bark. Similarly, Gemmatimonadetes, Bacteroidetes, and Deinococcus were clustered together, with the highest contents in seedling leaves and stems. Globally, the phylum with the highest abundance in the agarwood *A. sinensis* bark was Firmicutes, while the phylum with the lowest abundance was Proteobacteria.

With respect to the exophytic bacteria identified at the phylum level, young leaves and young branches clustered together; seeds, flowers, and bark clustered together; and leaves and white trunks clustered together. Similarly, the analysis of the species clustering tree revealed that Nitrospirae, Chloroflexi, Dependentiae, and Elusimicrobia were clustered together, with all present at their highest levels in seedling roots. Chlamydiae, Patescibacteria, and Verrucomicrobia also clustered together, exhibiting their highest abundances in seedling roots and brown mature stems. The phylum Gemmatimonadetes also displayed a relatively high abundance in brown mature stems. Among the phyla of the external bacteria detected in the young leaves and stems, Deinococcus had the highest abundance. Moreover, the abundances of bacterial phyla in the bark of *A. sinensis* were highly consistent with the results for endophytic bacteria.

At the genus level, the exophytic bacteria of young leaves and young branches were clustered into one group; those of leaves, branches, and trunks were clustered into one group; those of flowers and bark were clustered into one group; and those of white trunks and brown trunks were clustered into another group. Through the analysis of the species clustering tree, *Acinetobacter* and *Sphingobium* coclustered, and their abundances were the highest in the stems of *A. sinensis*, while *Novosphingobium*, *Burkholderia*, and *Cupriavidus* coclustered into one group, and they all were present at the highest abundances in young roots. Compared with other tissues, *Bacillus* had the highest abundance in *A. sinensis* bark, and among all tissues, *Curtobacterium* had the highest abundance in *A. sinensis* seeds.

For the endophytic fungi, at the genus level, young branches, bark, and agarwood bark all clustered into one group. One can speculate that these three groups cluster together because they all contain bark. Similarly, white trunks and brown trunks can be clustered into a single group. By analysing the species clustering tree, *Fonsecaea* and *Acrocalymma* clustered into one group, and both had the highest abundances in *A. sinensis* brown trunks. Similarly, *Peethambara*, *Kurtzmanomyces*, and *Ascotaiwania* clustered into one group, but all had the highest abundances in *A. sinensis* white trunks. *Ilyonectria*, *Mortierella*, *Cutaneotrichosporon*, and *Aspergillus* cluster into one group, and all have the highest abundances in healthy trunks (Du et al. 2022a, b). In contrast, *Strelitziana*, *Phaeophleospora*, *Pseudocercospora*, and *Symmetrospora,* which coclustered, all exhibited the highest abundances in mature leaves. Moreover, *Toxicocladosporium* was most abundant in mature branches. An analysis of the endophytic fungi at the phylum level revealed that Mortierellomycota, Choanoflagellata, Cnidaria, and Chytridiomycota clustered into a single group, and all had the highest abundances in the seeds. The phylum Ascomycota had the highest abundance in young leaves and the lowest abundance in the brown trunks of *A. sinensis*. Basidiomycota had the lowest abundance in young stems and the highest abundance in the white trunks of *A. sinensis*. Glomeromycota had the highest abundance in healthy tree bark, while Mucoromycota had the highest abundance in young roots.

For the exophytic fungi, the cluster analysis at the phylum level revealed that young leaves, leaves and branches clustered together, and seeds, flowers, bark and trunks clustered together. Finally, white trunks, brown trunks, and agarwood bark cocluster together, which is perhaps unsurprising since they all represent mature *A. sinensis* tissues with galls. As such, the community similarity is consistent with the biological status of the plant. According to the analysis of the species clustering tree, Chytridiomycota and Rozellomycota clustered together, and both had relatively high abundances in young roots. Similarly, Mortierellomycota, Olpidiomycota, Ascomycota, and Glomeromycota clustered together, with higher abundances in the galls of *A. sinensis*; Olpidiomycota had the highest abundance in white trunks; Glomeromycota was most highly abundant in brown trunks; and Mortierellomycota had the highest abundance in agarwood bark (Du et al. 2022a, b). Basidiomycota exhibited the highest abundance in the young stems but the lowest abundance in the adult bark. For the exophytic fungi, the analysis of the sample clustering tree at the genus level revealed that young leaves and young branches were clustered together, while flowers, leaves, and branches were clustered together, and white trunks, brown trunks, and agarwood bark were clustered together. These three groups are composed of seedlings, healthy adults, and galls containing *A. sinensis* tissues, respectively, and thus their community similarities are high. Through an analysis of the species clustering tree, *Fonsecaea*, *Acrocalymma*, *Peethambara*, and *Debaryomyces* were clustered together, with higher contents in the galls of *A. sinensis*. Similarly, *Mortierella*, *Aspergillus*, *Coniochaeta*, and *Rhizophagus* clustered together, with higher contents in trunk tissues, and *Aspergillus* had the highest content in healthy trunks, which is consistent with previous research results (Liu et al. 2022a, b, c, d). Moreover, *Dioszegia*, *Strelitziana*, *Phaeophleospora*, *Pseudocercospora*, and *Symmetrospora* clustered together and were mainly present in flowers, leaves, and branches; *Dioszegia* and *Strelitziana* were present in the greatest amounts in branches; and *Phaeophleospora*, *Pseudocercospora*, and *Symmetrospora* exhibited the greatest abundance in leaves. *Colletotrichum*, *Setophoma*, *Erythrobasidium*, and *Uwebraunia* clustered together, mainly in young leaves and stems, with *Uwebraunia* having the highest content in young leaves and *Colletotrichum*, *Setophoma*, and *Erythrobasidium* having the highest contents in young stems. In contrast, the highest content of *Periconia* was detected in the seeds.

### Analysis of the differences in the microbial community composition in different tissues

**Fig. 3:**
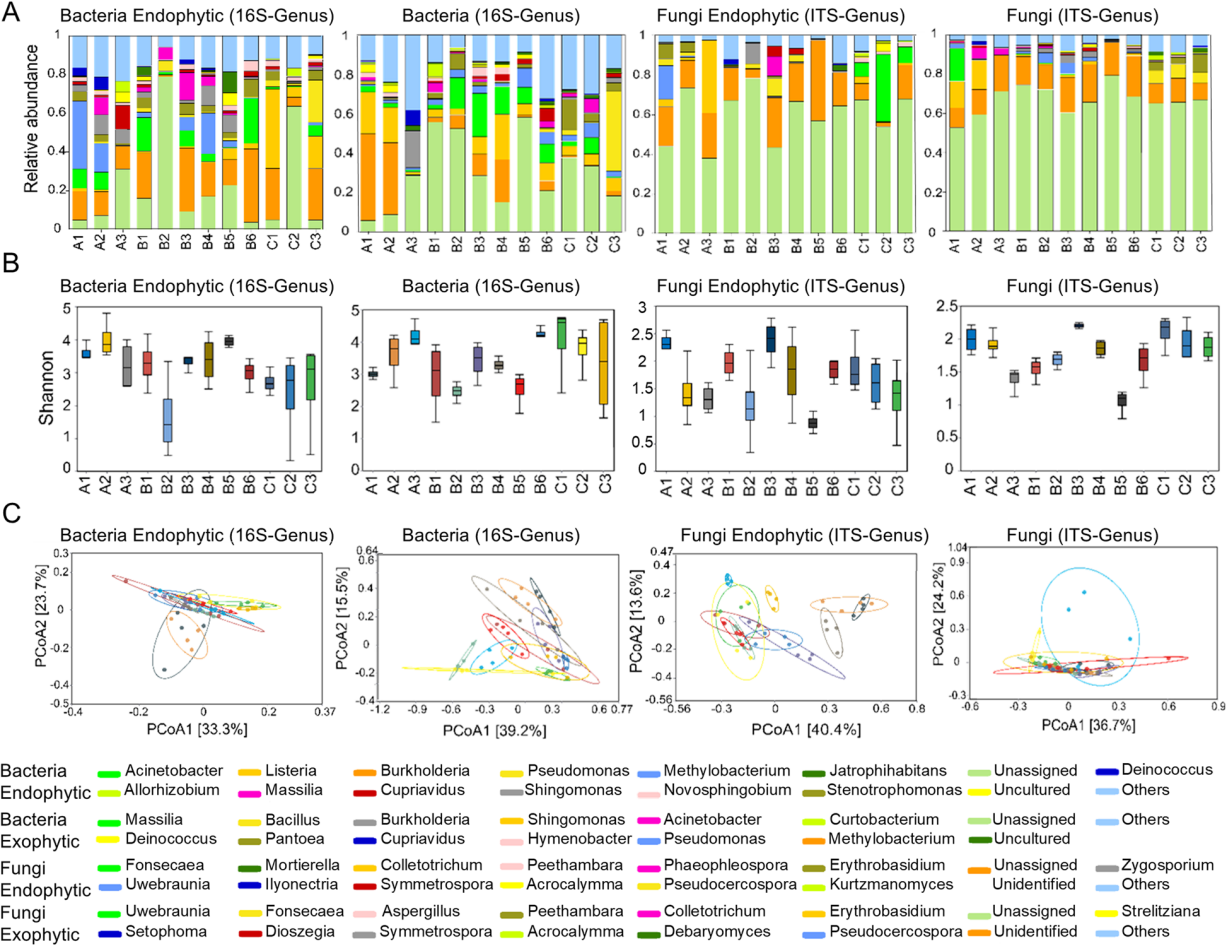
Analysis of the microbial community compositions and diversities in various *A. sinensis* tissues at the genus level. **A** The bacterial community compositions in different tissues. The horizontal axis represents the sample names, and the vertical axis represents the relative abundance. The stacked bars show the relative abundance at each taxonomic level, and the colours are used only to differentiate between different tissues. **B** Analysis of the difference in the Shannon index among different plant tissues. The Shannon index is shown on the vertical axis, and sample names are shown on the horizontal axis. The median, dispersion, maximum, and minimum values of the species diversity within each group can be visually observed. The Kruskal‒Wallis rank-sum test (using the kruskal test function in R) was used to evaluate the differences in the diversity indices between different groups. **C** Principal coordinate analysis (PCoA) of the microbial communities in various tissues. Each point represents a sample, and points with the same colour are from the same group, while points with different colours represent different sample groups. The closer the distance between two points is, the smaller the difference in community composition between them, indicating that the community compositions of the sample groups are more similar. PCoA1 and PCoA2 are the two principal coordinate components, with PCoA1 representing the principal coordinate component that explains the largest possible amount of variance in the data and PCoA2 representing the principal coordinate component that explains the largest proportion of the remaining variance. The first group consisted of 5-month-old *A. sinensis* seedlings (young leaf/A1, young branch/A2, young root/A3); the second group consisted of healthy 7-year-old *A. sinensis* (seed/B1, flower/B2, leaf/B3, branch/B4, bark/B5, trunk/B6); and the third group consisted of mature *A. sinensis* with agarwood (white trunk/C1, brown trunk/C2, agarwood bark/C3)

According to the compositions of the *A. sinensis* tissue communities (Fig. 3A; Figure S1A), the endophytic bacteria were divided into three main phyla: Proteobacteria, Firmicutes, and Actinobacteria. Proteobacteria were relatively abundant in all tissue communities and were the dominant phylum. This phylum also includes some rhizobium genera that are symbiotic with plants, as well as *Rhodocyclus* and *Rubrivivax*, which are related to photosynthesis (Mohamed et al. 2014a, b; Tibpromma et al. 2021). Firmicutes were present mainly in the bark, trunks, white trunks, brown trunks, and agarwood bark tissues and appeared to be particularly important in aromatic wood tissues. Actinobacteria had the lowest relative abundance in the flowers but was among the three most abundant phyla in the other tissues.

The 15 most abundant endophytic bacterial genera were mainly from the aforementioned three phyla. For example, the genera *Burkholderia*, *Methylobacterium*, *Pseudomonas*, *Stenotrophomonas*, *Sphingomonas*, *Massilia*, *Cupriavidus*, *Novosphingobium*, and *Allorhizobium* all belong to the phylum Proteobacteria taxonomically, while the genus *Listeria* belongs to the phylum Firmicutes, while the genus *Acinetobacter* belongs to the phylum Actinobacteria. *Burkholderia* was three times more abundant in stem tissues than in nonwood aromatic tissues, while *Listeria* was four times more abundant in white trunk samples than in brown trunk samples. *Acinetobacter* ranked second in the trunk community but accounted for only approximately 1.5% of the white trunk community and even less in the brown trunk community. Additionally, *Allorhizobium* was significantly expressed only in young roots and brown trunks, and thus *Acinetobacter* and *Allorhizobium* may be related to the formation of agarwood.

The exogenous bacteria can be divided into four main phyla: Proteobacteria, Actinobacteria, Firmicutes, and Bacteroidetes. Proteobacteria was the most abundant phylum of this class, while Firmicutes accounted for the highest proportion in agarwood bark compared with other tissues. An assessment of the characteristics of the exogenous bacteria revealed the following: (i) *Methylobacterium* was the predominant exogenous bacterial genus in young leaves and young branches; (ii) *Bacillus* had a relatively higher abundance in agarwood bark, which may be due to various external stimuli received by *A. sinensis* wood; *Bacillus* is a stress-resistant bacterium that can secrete spores that have a special resistance to adverse conditions when exposed to external harm; and (iii) *Acinetobacter* had a relative abundance in brown trunks that was approximately three times greater than that in white trunks and is an opportunistic pathogen that maintains a good balance between normal microbial flora and hosts through factors such as nutrient competition and metabolic product constraints. However, when this balance is disrupted, some bacteria in the original normally nonpathogenic microbial flora can become pathogenic.

From the perspective of endophytic fungi, the phyla that dominated in terms of their relative abundances in the fungal community were Ascomycota, Basidiomycota, and Mortierellomycota. Ascomycota was the dominant group in all tissues and exhibited the highest abundance in brown trunks. The relative abundance of Mortierellomycota was significantly higher in the trunks than in the other tissues. In the brown trunks, the relative abundance of Basidiomycota was approximately half that in the white trunks and trunks. Most of the genera with high relative abundances of endophytic fungi in various tissues belong to these three phyla. The genera *Colletotrichum*, *Fonsecaea*, *Uwebraunia*, *Acrocalymma*, *Phaeophleospora*, *Zygosporium*, and *Ilyonectria* all belong to the phylum Ascomycota; the genus *Mortierella* belongs to the phylum Mortierellomycota; and the genera *Kurtzmanomyces* and *Erythrobasidium* belong to the phylum Basidiomycota. The proportions of these genera in various tissues also differed. The relative abundance of *Colletotrichum* was higher in young roots than in other tissues. *Fonsecaea* was a dominant species in white trunks, brown trunks, and agarwood bark, especially in brown trunks, which are the main sites for producing agarwood. *Erythrobasidium* was more dominant in young leaves and young branches than in other tissues. *Uwebraunia* was a dominant fungal genus in young leaves. In contrast, *Ilyonectria* was the dominant fungal genus in seeds, *Zygosporium* was the dominant fungal genus in flowers, *Phaeophleospora* was the dominant fungal genus in leaves, *Mortierella* was the dominant fungal genus in trunks, and *Acrocalymma* was the dominant fungal genus in brown trunks.

From the perspective of ectomycorrhizal fungi, the phyla that dominated in terms of their relative abundances in the fungal community were Ascomycota, Basidiomycota, and Mortierellomycota. Ascomycota was the dominant group in all tissues and was more evenly distributed in each tissue. Similarly, Basidiomycota was more dominant in the seedling tissues of *A. sinensis*. In contrast, Mortierellomycota was dominant in the agarwood tissue of *A. sinensis*. The ectomycorrhizal fungal genera with relatively high abundances were mainly distributed in two phyla, Ascomycota and Basidiomycota. The genera *Fonsecaea*, *Peethambara*, *Colletotrichum*, *Uwebraunia*, *Acrocalymma*, *Pseudocercospora*, and *Debaryomyces* belong to the phylum Ascomycota; the genera *Erythrobasidium*, *Symmetrospora*, and *Dioszegia* belong to the phylum Basidiomycota. The distributions of these ectomycorrhizal fungi at the genus level were characteristic of each tissue. *Erythrobasidium* was mainly present in young leaves and young branches. *Uwebraunia* mainly occurred on young leaves. This distribution pattern is consistent with that of the endophytic fungi. Colletotrichum was more dominant in young branches and young roots (Ma et al. 2021), whereas *Pseudocercospora* and *Symmetrospora* were the dominant genera in flowers, leaves, and branches. *Fonsecaea* was distributed in white trunks, brown trunks, and agarwood bark, with the highest proportion in brown trunks and the lowest proportion in agarwood bark, but the differences were not particularly significant. *Peethambara* was also distributed in white trunks, brown trunks, and agarwood bark, with the lowest proportion occurring in brown trunks and the highest proportion occurring in agarwood bark. *Acrocalymma* occupied large proportions of white trunks, brown trunks and agarwood bark and was most common in the trunks. *Debaryomyces* was only significantly abundant in agarwood bark.

### The diversity of the species communities differs in different growth stages and tissues

We conducted an intergroup analysis using an alpha diversity index based on the Shannon index to clarify the diversity of the species in the communities with different organizations (Fig. 3B; Figure S1B). The box plots visually display the median, dispersion, maximum, and minimum values of the species diversity within each group. Moreover, the Kruskal‒Wallis rank sum test was used to assess whether significant differences in the diversity indices of the *A. sinensis* groups occurred at different stages (Li et al. 2023).

At the genus level, young branches and bark had greater diversities of endophytic bacterial species than did the other groups, while young roots, flowers, branches, brown trunks and agarwood bark displayed greater diversities of endophytic bacterial species within each group; young leaves, leaves, and bark displayed lower diversities. However, the overall diversity of the endophytic bacterial species in *A. sinensis* seedlings was greater than that in adult *A. sinensis* plants. In addition, the diversity of the exogenous bacterial species was greater than that of the endophytic bacterial species in all groups, and the diversity of the exogenous and endogenous bacterial species in the bark tissue of *A. sinensis* was much greater than that of the endophytic bacteria. However, the richness of the endogenous and exogenous bacterial species in the brown trunks of *A. sinensis* was much lower than that of the endophytic bacteria. Moreover, the diversity of the exogenous bacterial species in *A. sinensis* was significantly greater than that in healthy *A. sinensis*.

At the phylum level, the diversity of the endophytic bacterial species in young stems was much greater than that in other tissues. However, the richness of the endophytic bacterial species within each group was greater in the brown trunk tissues of *A. sinensis*. The diversity of the exogenous bacterial species was also greater than that of the endophytic bacterial species in all tissues, but the richness of both the exogenous and endogenous bacterial species within each group was not as high as that of the endophytic bacteria. Greater diversity of exogenous bacterial species was detected in the young roots, while the groups with the greatest diversity of both exogenous and endogenous bacterial species were the seeds, white trunks and brown trunks.

At the genus level, the diversity of endophytic fungi was much greater than that of exophytic fungi, and the richness of endophytic fungi within each group was also greater than that of exophytic fungi. However, the diversity of the exophytic fungi in *A. sinensis* was greater than that of the endophytic fungi. According to the Shannon index of the endophytic fungi in the trunks, the richness decreased in the order of healthy trunks > white trunks > brown trunks, while the richness of the exophytic fungi decreased in the order of white trunks > brown trunks > healthy trunks. At the leaf level, the fungal diversity of adult leaves was considerably greater than that of young leaves, whether endophytic or exophytic, and the diversity of adult branches was also greater. However, a significant difference in fungal richness was observed between healthy adult bark and other tissues, and it was significantly lower than that in other tissues (Liu et al. 2019).

At the phylum level, the diversity of the exophytic fungi was still significantly greater than that of the endophytic fungi, but the within-group diversity of the endophytic fungi was greater than that of the exophytic fungi. The brown trunks of *A. sinensis* were tissues with greater diversities of both endophytic and exophytic fungi, and the richness of the exophytic fungi within these tissues was the highest. According to the Shannon index for the endophytic fungi in all trunk tissues, the richness decreased in the order of healthy trunks > white trunks > brown trunks; however, the within-group richness of the brown trunks was still relatively high. However, the richness of the exophytic fungi in brown trunks was relatively high both in terms of the Shannon index and within-group diversity.

### Community compositions differ among different growth stages and different tissues

Principal component analysis (PCA) of the community composition of each tissue type at the genus/phylum level was conducted using R software (Fig. 3C; Figure S1C). The potential principal components that affect the differences in community composition among tissues were identified. As shown in Fig. 3C and Figure S1C, at the phylum level, little difference in the bacterial communities was observed among all tissues except for the agarwood *A. sinensis* tissues, indicating a significant difference in the community composition between the agarwood *A. sinensis* tissues and the other plant tissues at different growth stages. In addition, when comparing the bacterial community compositions of the three types of trunks, the bacterial community composition of healthy trunks was highly similar to that of both brown and white trunks. However, the healthy trunks exhibited lower diversity than did the brown and white trunks. The community compositions of other tissues were relatively similar, with highly similar bacterial community structures (Wang et al. 2022a, b).

At the genus level, the compositions of the endophytic bacterial communities in different *A. sinensis* tissues, except for the roots, were similar to those of the seedlings. In mature *A. sinensis* trees, the compositions of the endophytic bacterial communities in different tissues were more similar, but the overall differences among tissues were not particularly significant. However, the compositions of the external bacterial communities in the leaves and young stems/branches of the seedlings were more similar, and partial similarity was observed between the external bacterial community compositions of the young and mature branches. Nonetheless, the external bacterial community compositions of most mature plant tissues were more similar. The ellipsoid distribution range of *A. sinensis* tissues was greater in mature plants than in seedlings, and the ellipsoid distribution range of agarwood *A. sinensis* tissues was greater than that in healthy *A. sinensis* tissues.

At the genus level, the compositions of the endophytic fungi in most *A. sinensis* tissues, except for young roots, bark, brown trunks, and agarwood bark, were similar. Although the structures for the composition of the exogenous fungi in each tissue were somewhat similar, the overall structures of the compositions for the three major groups, namely, *A. sinensis* seedlings, agarwood *A. sinensis*, and healthy *A. sinensis*, were more similar.

At the phylum level, the compositions of the endophytic fungi in various tissues were more similar, but the elliptical interval distribution range of healthy adult *A. sinensis* trees was greater. Based on the three major categories of *A. sinensis* seedlings, namely, agarwood *A. sinensis* and healthy *A. sinensis*, the structures of the compositions of both the endophytic and exogenous fungi were more similar within each group. In addition, the agarwood *A. sinensis* agarwood composition was not only similar but also had a broader distribution range.

**Fig. 4:**
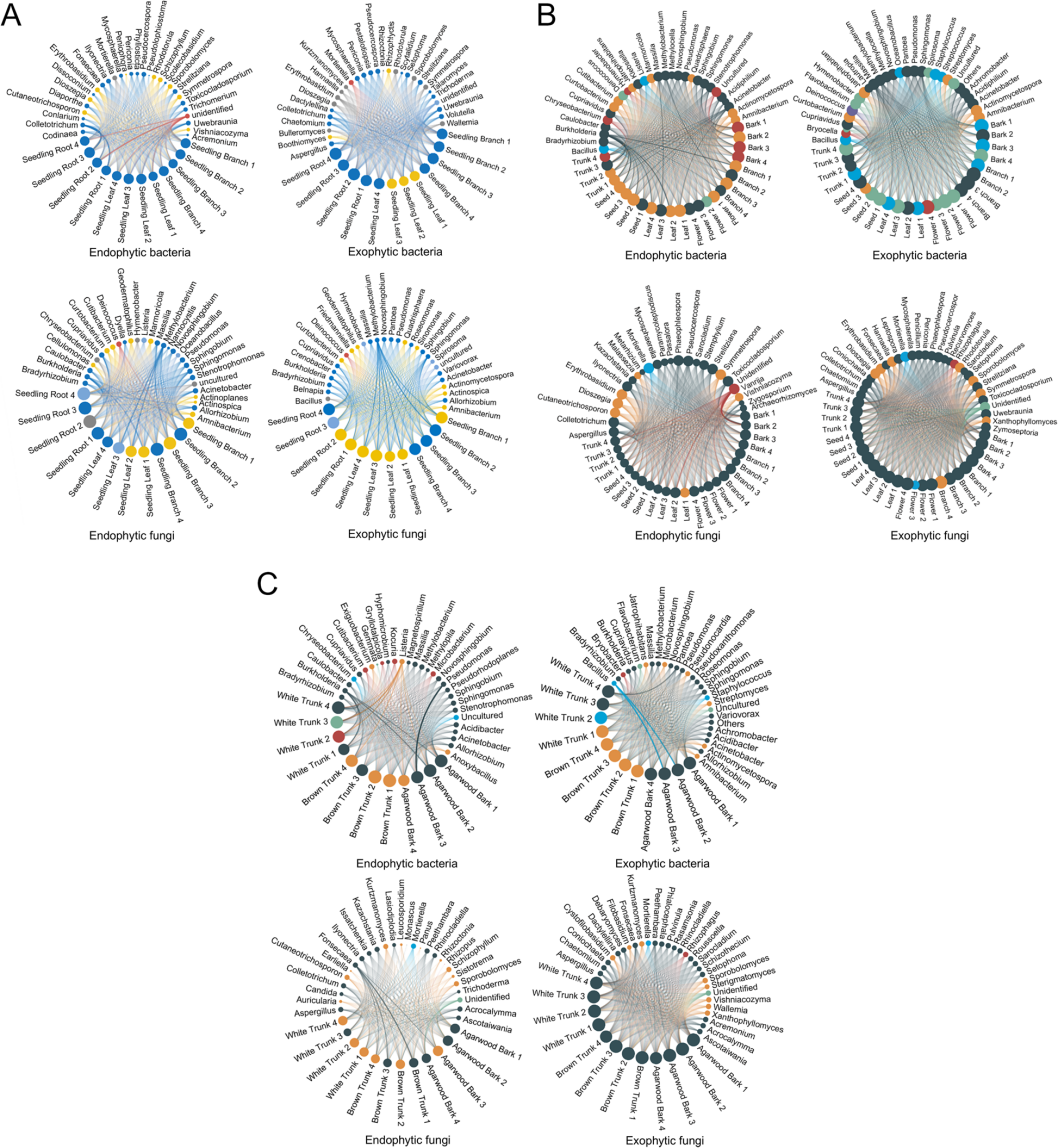
Chord diagrams of the bacterial and fungal communities at different growth stages of *A. sinensis*. **A** Chord diagram showing the collinearity of fungal/bacterial communities in *A. sinensis* seedlings. Four replicates of each tissue sample were used. The analysis encompassed four perspectives: endophytic bacteria, exophytic bacteria, endophytic fungi and exophytic fungi. Two types of nodes are present in the chord diagrams: species nodes and sample nodes. If a species appears in a sample, a line is drawn between the species and the sample. The colours of the sample nodes follow the species nodes with the highest contributions. The sizes of all nodes are proportional to all their lines. The thickness and transparency of the lines are related to the species abundance. The greater the relative abundance of the species in the sample is, the thicker and more pronounced the lines between them are, and vice versa. **B** Chord diagram showing the collinearity of the fungal/bacterial communities in adult *A. sinensis*. Four replicates were performed for each tissue. The analysis encompassed four perspectives: endophytic bacteria, exophytic bacteria, endophytic fungi and exophytic fungi. Two types of nodes are present in the chord diagram: species nodes and sample nodes. If a species appears in a sample, a line is drawn between the species and the sample. The colours of the sample nodes follow the species nodes with the highest contributions. The sizes of all nodes are proportional to all their lines. The thickness and transparency of the lines are related to the species abundance. The greater the relative abundance of the species in the sample is, the thicker and more pronounced the lines between them are, and vice versa. **C** Chord diagram showing the collinearity of the fungal/bacterial communities in *A. sinensis* agarwood. Four replicates were performed for each tissue. The analysis encompassed four perspectives: endophytic bacteria, exophytic bacteria, endophytic fungi and exophytic fungi. Two types of nodes are present in the chord diagram: species nodes and sample nodes. If a species appears in a sample, a line is drawn between the species and the sample. The colours of the sample nodes follow the species nodes with the highest contributions. The sizes of all nodes are proportional to all their lines. The thickness and transparency of the lines are related to the species abundance. The greater the relative abundance of the species in the sample is, the thicker and more pronounced the lines between them are, and vice versa

Figure 4 provides a more intuitive reflection of the proportional compositions of the dominant species in each group while also illustrating the distribution proportions of the dominant species across different groups to further analyse the differences in the bacterial and fungal communities among different tissues and among different developmental stages within the same tissue. Within the seedling tissues, the dominant species composition of the external bacteria was more complex than that of the internal bacteria, whereas the dominant species composition of the external fungi was not as high as that of the internal fungi (Fig. 4A). In the tissues of mature plants, the dominant species composition of the external bacteria was relatively more complex than that of the internal bacteria, and simultaneously, the dominant species composition of the external fungi was also more complex than that of the internal fungi (Fig. 4B). Regarding the agarwood-producing plants, the dominant species composition of the internal bacteria/fungi within each tissue was more complex than that of the external bacteria/fungi. However, the relative abundance of the dominant species of the external fungi was greater than that of the internal fungi (Fig. 4C). A comparative analysis of the different growth stages within the same *A. sinensis* tissue revealed that, except for leaves, the dominant species compositions of the external bacteria and external fungi in the bark, trunks, and branches were more complex than those of the internal bacteria and internal fungi. Additionally, the dominant species compositions of internal bacteria in young leaves and external bacteria in leaves were even more complex. Furthermore, the dominant species composition of the external fungi in leaves was more complex than that of the internal fungi (Figure S2).

## Discussion

### Representative bacterial and fungal genera among *A. sinensis* tissues

**Fig. 5:**
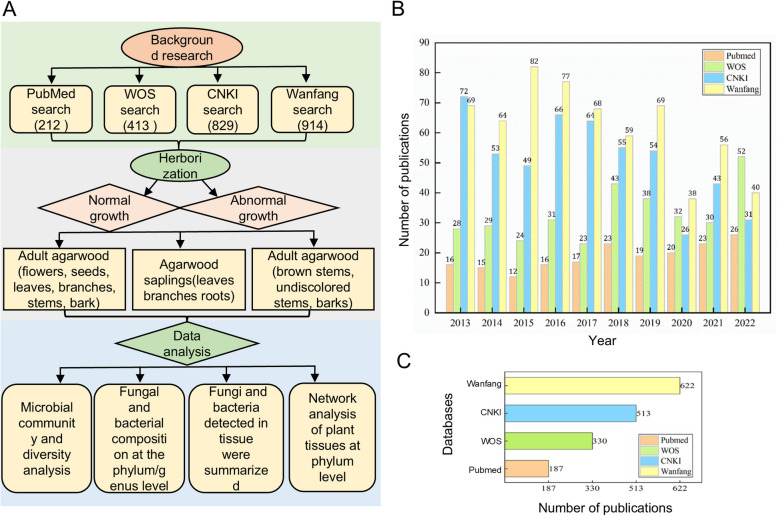
Background on *A. sinensis* research and its conceptual framework. **A** Overall flow chart of the research design. The overall idea of this paper is to first investigate the background literature, then collect samples, and finally analyse the experimental data. **B** Number of articles on *A. sinensis* published in each database each year. The figure shows a statistical analysis of the literature search results from Chinese and English databases, such as PubMed, Web of Science, China National Knowledge Infrastructure and Wanfang Database, up to December 31, 2022. **C** Total number of articles on *A. sinensis* published in each database over the past decade. The figure is a total of the articles on *A. sinensis* retrieved from four databases (PubMed, Web of Science, China National Knowledge Infrastructure and Wanfang) that were published in the last ten years

A literature search was conducted on published agarwood research using the keyword "*Aquilaria sinensis*" in the four most commonly used Chinese and English databases: PubMed, Web of Science (WOS), China National Knowledge Infrastructure (CNKI), and the Wanfang Database. The total number of articles published in these databases over the past decade (January 1, 2013, to December 31, 2022) was counted, and the annual publication trends were compared. Overall, the number of "*Aquilaria sinensis*"-related articles in the English databases showed an upwards trend, while in the Chinese databases, these numbers exhibited a declining trend followed by an increase and then another decline. As *A. sinensis* is an important woody plant, enhancing its relevant data and information is needed. Through an analysis of prior research statistics, the authors were able to discern the limitations and unresolved issues pertaining to the existing studies on *A. sinensis*. Therefore, tissues were collected from specific growth stages, and further analysis of the sequencing results of these samples was performed (Fig. 5).

Most of the current research on *A. sinensis*, commonly known as agarwood, revolves around the trunk, with only a few studies focusing on the leaves, and limited information is available on other tissues of *A. sinensis*. Based on the results of our analyses (Figs. 2, 3, 4 and S1-S2), we can summarize the highly abundant bacterial genera and fungal genera among these 12 tissues.

In 2014, Gao et al. studied the distribution of endophytic fungi in the leaves of *A. sinensis*. They found that these fungi were primarily located in the spongy and phloem tissues, with the highest abundance in the cambium layer of *A. sinensis*. *Collectotrichum* was identified as the dominant species in both healthy *A. sinensis* leaves and agarwood-producing *A. sinensis* leaves. Additionally, Gao et al. speculated that *Fusarium* may induce resin formation in *A. sinensis*(Gao et al. 2014). Furthermore, our research revealed the significance of certain bacteria in both the seedling and adult leaves of *A. sinensis*. Specifically, *Burkholderia*, *Acinetobacter*, *Methylobacterium*, and *Sphingomonas* were found to be important in both stages. In the seedling leaves, *Deinococcus* and *Stenotrophomonas* were the dominant bacteria, whereas *Massilia* and *Hymenobacter* were dominant in the adult leaves. Moreover, when examining the fungi present in *A. sinensis* leaves, we observed that *Erythrobasidium* and *Uwebraunia* were the main fungi in seedling leaves, while *Pseudocercospora*, *Phaeophleospora*, and *Symmetrospora* were dominant in adult leaves. These findings contribute to further research on agarwood and provide insights into the distributions of endophytic fungi and bacteria in *A. sinensis*.

In addition, we compiled a summary of the dominant genera in several understudied tissues of *A. sinensis*, including young roots, flowers, and seeds. For the young roots, the predominant bacterial genera were *Burkholderia*, *Sphingomonas*, *Cupriavidus*, *Novosphingobium*, and *Allorhizobium*. The primary fungal genus detected was *Colletotrichum*. The primary bacterial genera that were detected in the seeds were *Burkholderia*, *Acinetobacter*, *Stenotrophomonas*, *Jatrophihabitans*, and *Cupriavidus*. The main fungal genera that were identified were *Ilyonectria*, *Pseudocercospora*, *Symmetrospora*, and *Periconia*. For the flowers, the dominant bacterial genera observed were *Pseudomonas*, *Massilia*, *Methylobacterium*, and *Pantoea*. The main fungal genera detected were *Pseudocercospora* and *Zygosporium*. Furthermore, most of the current research on *A. sinensis* has focused on its healthy stems and agarwood-producing stems, and a comprehensive understanding of its complete growth process is lacking. Therefore, we conducted additional research on the tissues of seeding stems and adult *A. sinensis* branches. In the seeding stems, we found that the main bacteria were *Burkholderia*, *Acinetobacter*, *Methylobacterium*, *Sphingomonas*, and *Massilia*, while the main fungi were *Erythrobasidium*, *Pseudocercospora*, and *Colletotrichum*. For the adult *A. sinensis* branches, the predominant bacteria were *Burkholderia*, *Methylobacterium*, *Sphingomonas*, *Massilia*, and *Hymenobacter*, and the primary fungi identified were *Pseudocercospora*, *Symmetrospora*, and *Dioszegia*. These findings provide valuable insights into the microbial compositions of different *A. sinensis* tissues, highlighting the understudied aspects of this plant species.

### A comparison between healthy tissues and agarwood reveals possible microbial genera related to agarwood formation

The stems are the main site of agarwood formation, and thus most current research has focused on observing the effects of different injury methods on agarwood formation in the stems. Wang et al. explored the bacterial community structure of agarwood samples that were generated through various treatments, including healthy agarwood, liquid fermentation agarwood, insect attack agarwood, and drilling agarwood (Wang et al. 2022a, b). They observed that the main bacteria in healthy agarwood were *Amnibacterium* and *Delftia*. In this study, we investigated the bacterial genera that were present in the white trunks of agarwood. We identified *Burkholderia*, *Listeria*, *Stenotrophomonas*, *Sphingomonas*, *Novosphingobium*, and *Pantoea* as the main bacterial genera. Additionally, the main fungal genera that were detected in the white trunks were *Colletotrichum*, *Fonsecaea*, *Peethambara*, *Kurtzmanomyces*, and *Acrocalymma*.

In contrast, in drilling agarwood, the predominant bacteria were *Actinoplanes*, *Sphingomonas*, *Bordetella*, and *Sphingobacterium*. *Pelagibacterium* and *Methylovirgula* are the primary bacteria found in fermentation liquid agarwood, while *Cellulomonas*, *Sphingobium*, and *Aeromicrobium* are the predominant bacteria found in insect attack agarwood (Wang et al. 2022a, b). Furthermore, these authors found that when the trunks were not exposed to air, the bacterial abundance significantly decreased. Agarwood-producing *A. sinensis* exhibited greater bacterial diversity, consistent with our findings. However, Wang et al. considered *Sphingomonas* to be the main bacteria in drilling agarwood, whereas in this study, we speculate that it may be a bacterium that is widely present in *A. sinensis* branches and stems since we observed its presence in seedling stems, mature branches, healthy adult stems, and healthy adult agarwood-producing stems. *Sphingobium* was the main bacterium in insect-attacked agarwood reported in Wang's study (Wang et al. 2022a, b; Zhang et al. 2020), but in the current research, it primarily appeared in agarwood-producing *A. sinensis* stems.

In previous studies, the dominant bacteria in healthy trunks were *Amnibacterium* and *Delftia*. The dominant bacteria in agarwood subjected to different treatment methods were *Actinoplanes*, *Bordetella*, *Sphingobacterium*, *Pelagibacterium*, *Methylovirgula*, *Cellulomonas*, and *Aeromicrobium* (Du et al. 2022a, b; Wang et al. 2022a, b). A total of 15 possible genera related to agarwood were identified in our current study, including *Listeria*, *Pseudomonas*, *Acinetobacter*, *Sphingobium*, *Bacillus*, *Fonsecaea*, *Acrocalymma*, *Peethambara*, *Kurtzmanomyces*, *Ascotaiwania*, *Burkholderia*, *Allorhizobium*, and *Debaryomyces*.

The fungi that were identified were *Fonsecaea*, *Acrocalymma*, *Peethambara*, *Kurtzmanomyces*, *Ascotaiwania*, and *Debaryomyces*. The bacteria identified were *Listeria*, *Pseudomonas*, *Acinetobacter*, *Sphingobium*, *Bacillus*, *Burkholderia*, and *Allorhizobium*. *Listeria* and *Peethambara* are both plant pathogens (Canlı et al. 2016; Lombard et al. 2016; Truong et al. 2021). *Kurtzmanomyces* and *Ascotaiwania* are commonly found in plants, with *Kurtzmanomyces* often present in various forms (Cui et al. 2021; Zhang et al. 2019). *Acrocalymma*, *Sphingobium*, *Allorhizobium*, and *Bacillus* are associated with various plant stress responses and can induce defence mechanisms in plants. Additionally, *Sphingobium* is a beneficial bacterium for plants, helping them resist pathogen attacks and promote healthy growth (Csorba et al. 2022; He et al. 2021; Li et al. 2022; Tzipilevich et al. 2021; Zhang et al. 2022). *Fonsecaea* is a pathogen that is primarily harmful to humans, while *Pseudomonas* can be used for biocontrol because it enhances plant immunity and promotes growth (Oni et al. 2022; Villena et al. 2020). *Acinetobacter* is a naturally occurring antibacterial compound that has been isolated from plants, and *Debaryomyces* can serve as an antibacterial and biocontrol agent (Cadelis et al. 2023; Hammami et al. 2022; Shruthi et al. 2022).

In our study, by comparing the healthy trunks and brown trunks of agarwood, we identified common dominant bacteria, including *Burkholderia*, *Sphingomonas*, *Massilia*, *Pseudomonas*, and *Acinetobacter*. The brown trunk of agarwood had unique dominant bacteria, namely, *Listeria* and *Allorhizobium*, while the healthy trunks had *Stenotrophomonas* and *Novosphingobium* as the unique dominant bacteria. Furthermore, the main fungal genera detected in the brown trunk of agarwood were *Fonsecaea*, *Peethambara*, and *Acrocalymma*, while in the healthy trunk, they were *Mortierella*, *Symmetrospora*, and *Aspergillus*. Another important tissue in *A. sinensis* is the bark, which serves as a vital pathway for nutrient transport in woody plants. Therefore, characterizing agarwood-producing *A. sinensis* bark and healthy *A. sinensis* bark is also crucial. We found that the main dominant bacteria in healthy bark included *Burkholderia*, *Sphingomonas*, *Jatrophihabitans*, *Pseudomonas*, *Deinococcus*, and *Massilia*. In agarwood bark, the main dominant bacteria were *Burkholderia*, *Listeria*, *Pseudomonas*, *Sphingomonas*, *Stenotrophomonas*, and *Bacillus*. Regarding the fungal genera in both cases, agarwood bark had a significantly greater number of dominant fungi than healthy bark, with only *Pseudocercospora* being significantly dominant in the healthy bark. However, agarwood bark exhibited dominant fungal genera such as *Fonsecaea*, *Peethambara*, and *Debaryomyces*. Therefore, incense formation may be regulated by a variety of factors rather than by simple fungal infection. Furthermore, environmental factors exert significant influences on both microbial communities and agarwood formation. Studies have indicated that during the process of agarwood formation, the rates of discolouration following damage in the rainy season were three times greater than those in the dry season, which is possibly attributable to the more favourable environmental conditions of high humidity and reduced light intensity, which facilitate microbial activity, as well as enhanced spore dispersal in windy and aqueous environments. Moreover, the predominance of humid and warm climates in agarwood-forming regions may also be pertinent to these phenomena.

To summarize, different treatment methods can be inferred to result in variations in the bacterial compositions within the stems. However, these different treatment methods also mutually influence each other, as physical trauma can potentially lead to insect attacks. Therefore, under conditions without external influences, agarwood formation is mostly induced by a combination of multiple injury mechanisms. Furthermore, the formation of agarwood in *A. sinensis* is influenced by various bacteria, and the bacteria that are secreted during the agarwood formation process are mostly associated with defence mechanisms.

### Biosynthesis of the fragrance and active constituents of *A. sinensis* may be affected by its commensal microbiome

Previous studies have shown that the main constituents of agarwood resin are sesquiterpenes and chromones. In this study, the agarwood stems were divided into two main parts: a brown section and a white section. However, the agarwood trunks of *A. sinensis* can be further divided into distinct layers, including the decay layer, decay–agarwood transition layer, agarwood layer, agarwood–normal transition layer, and normal white layer. Moreover, Liu et al. found that among these five layers, only the cells in the agarwood–normal transition layer and the normal white layer were viable, while the majority of cells in the other layers were already dead. Additionally, their research revealed that sesquiterpenes and chromones were mainly distributed in the decay–agarwood transition layer and agarwood layer. Furthermore, the abundances of sesquiterpenes and chromones showed negative correlations with fungal diversity. Specifically, the fungus *Phaeoacremonium rubrigenum* promoted the accumulation of sesquiterpenes and chromones in agarwood and exhibited a strong ability to induce sesquiterpene biosynthesis. Fungus-induced sesquiterpene biosynthesis primarily occurs through the mevalonate pathway, with high levels of phosphorylation activating upstream transcription factors to form a responsive network (Liu et al. 2022a, b, c, d; Zhang et al. 2014). In summary, during agarwood formation in *A. sinensis*, the richness of the endophytic bacterial community significantly increases, but the diversity of the endophytic fungi does not differ significantly. The bacterial community diversity in *A. sinensis* seedlings was greater than that in adult *A. sinensis* plants, but the difference in the fungal community diversity between seedlings and adult *A. sinensis* plants was not significant. Moreover, for both bacteria and fungi, the Shannon indices for the exogenous microorganisms were greater than those for the endogenous microorganisms. The main bacterial phyla in *A. sinensis* are Proteobacteria, Firmicutes, Actinobacteria, and Bacteroidetes; the main fungal phyla are Ascomycota and Basidiomycota. These findings are consistent with previous research (Lee et al. 2019; Wang et al. 2018). *Colletotrichum* is more prevalent in seedling tissues, and if cultivating *A. sinensis* seedlings is the goal, attention should be given to *Colletotrichum*. *Ilyonectria*, *Curtobacterium*, and *Periconia* are more prevalent in seeds, and if you want to learn about *A. sinensis* seeds, these genera should be the focus.

## Conclusions

In this study, the diversity of the endophytic bacterial species was greater during the seedling stage than during the healthy adult stage. However, the diversity of the exogenous bacterial species was greater during the agarwood *A. sinensis* stage than during the healthy adult stage. At the genus level, the abundances of the bacterial communities followed the pattern of the agarwood *A. sinensis* stage > healthy adult stage > seedling stage. The bacterial community composition structures of various tissues in the healthy adult stage were more similar, while the external fungal community composition structures in each stage (e.g., seedlings, agarwood, and healthy adults) of *A. sinensis* were more similar. At the phylum level, the internal fungal community composition structures of different tissues also showed greater similarity. *Listeria*, *Kurtzmanomyces*, and *Ascotaiwania* were highly abundant in the white trunks of agarwood, while *Acinetobacter*, *Sphingobium*, *Fonsecaea*, *Acrocalymma*, and *Allorhizobium* were highly abundant in the brown trunks of agarwood. The abundances of *Bacillus*, *Pseudomonas*, *Peethambara*, and *Debaryomyces* were higher in agarwood bark. These genus differences may play a crucial role in triggering the formation of *A. sinensis* agarwood, and further verification can be conducted for these bacteria. This resource article analyses the microbial community structures and changes from the seedling stage to the adult stage and subsequently to the agarwood stage, providing a solid theoretical foundation for improving the efficiency of *A. sinensis* agarwood production in the future. This study also supports better research on the growth and development of *A. sinensis* and serves as a scientific case study for the rational use of microorganisms in plant biotechnology.

### Experimental procedures

#### Sample collection and analysis

We collected three groups of *A. sinensis* tissues at different stages of growth. The first group consisted of 5-month-old *A. sinensis* seedlings (leaf/A1, branch/A2, root/A3), and the second group consisted of healthy 7-year-old *A. sinensis* tissues (seed/B1, flower/B2, leaf/B3, branch/B4, bark/B5, trunk/B6). The third group consisted of adult *A. sinensis* (white trunk/C1, brown trunk/C2, bark/C3); the method of inducing agarwood formation consisted of the drilling method that was used to cause physical trauma. All the mentioned tissues (A1/A2/A3/B1/B2/B3/B4/B5/B6/C1/C2/C3) were sampled, with four replicates for each tissue.

#### DNA extraction and sequencing

After genomic DNA extraction using a DNA extraction kit (Mabio, Guangzhou, China), the purity and concentration of the DNA were determined using a NanoDrop instrument (Thermo Scientific, Wilmington, DE). Genomic DNA was used as a template, and PCR amplification was performed using specific primers with barcodes and TaKaRa Premix Taq® Version 2.0 (TaKaRa Biotechnology Co., Dalian, China) based on the selected sequencing region. For fungal amplification, the sequences of the primers used were F: CTTGGGTCATTTAGAGGAAGTAA and R: GCTGCGTTCTTCATCGATGC. For bacterial amplification, the sequences of the primers used were F: AACMGGATTAGATACCCKG and R: ACGTCATCCCCACCTTCC. Subsequently, 1% agarose gel electrophoresis was used to assess the fragment lengths and concentrations of the PCR products (Bio-Rad, California, USA). Samples with main bands within the normal range (16S V5V7-1: 400 bp; ITS1-2: 250 bp) were selected for further experiments. The PCR products were then quantified using GeneTools Analysis Software (version 4.03.05.0, SynGene), and the required volume of each sample was calculated based on equal mass principles. The PCR products were mixed, and the mixed products were recovered using an E.Z.N.A.® Gel Extraction Kit (Omega, USA) to extract the target DNA fragments, which were eluted in TE buffer. Finally, the constructed amplicon library was sequenced using the Illumina Nova 6000 platform with PE250 sequencing (Ark Biosafety Technology (Guangzhou) Co., Ltd. Guangzhou, China).

#### Data analysis

The fastp tool (an ultrafast all-in-one FASTQ preprocessor, version 0.14.1, https://github.com/OpenGene/fastp) was used to perform sliding window quality trimming of the paired-end raw read data. Additionally, Cutadapt software was used to remove primers based on primer information at both ends of the sequences, resulting in quality-controlled paired-end clean reads. Next, usearch-fastq_merge pairs was utilized to filter out inconsistent tags based on the overlaps between PE reads, which yielded the raw concatenated sequences (raw tags). Subsequently, the fastp tool was used to perform sliding window quality trimming of the raw tag data, which generated effective concatenated fragments (clean tags). The OTUs were obtained based on the merged sequences using the UPARSE clustering method (Edgar 2013). Then, usearch-sintax was used to compare the representative sequences of each OTU with the SILVA (16S) and Unite (ITS) databases to obtain species annotation information (with a default confidence threshold of 0.8). The taxonomy results for the species annotations were divided into seven hierarchical levels: kingdom (L1), phylum (L2), class (L3), order (L4), family (L5), genus (L6), and species (L7). Finally, chloroplast- or mitochondria-annotated OTUs, as well as those that were unable to be annotated at the kingdom level, were excluded. This process resulted in the final count of effective tag sequences (No. of sequences) and the comprehensive OTU classification information table (OTU_table) for each sample, which were used for subsequent analysis. The community composition analysis, species abundance clustering analysis, and diversity analysis were conducted using R software. The Shannon index was calculated to assess the diversity of the samples, and alpha diversity refers to the analysis of intergroup differences conducted through both parametric tests (Kruskal‒Wallis rank sum test) and nonparametric tests (one-way ANOVA)(Callahan et al. 2016; Cui et al. 2011; Koetschan et al. 2010). The significance of the differences in the intergroup diversity indices of *A. sinensis* across different stages was assessed through the Kruskal‒Wallis rank-sum test. Principal coordinate analysis (PCoA) based on the OTU abundance table was conducted using the vegan package of R software and the distance algorithm (Bray_Curtis; Euclidean) for classifying the differences between samples. The species relationship map analysis, based on the OTU abundance table, involved calculating the Pearson and Spearman correlation coefficients in R software to determine the interactions between species within or between sample groups.

## Supplementary Information

Supplementary Material 1: Figure S1. Analysis of the microbial community compositions and diversities in various A. sinensis tissues at the phylum level. Figure S2. Chord diagrams of the bacterial and fungal communities were generated for different tissue sites. Figure S3. Clustering analysis of the microbial species abundance at the class, order, family, and species levels in various A. sinensis tissues.

## Data Availability

The raw data generated in our study are available from the National Center for Biotechnology Information under accession number PRJNA989284.

## Abbreviations

*A. sinensis*: *Aquilaria sinensis*
ITS: Internal transcribed spacer
WOS: Web of Science
CNKI: China National Knowledge Infrastructure
OUT: Operational taxonomic unit
PCA: Principal component analysis
PCoA: Principal coordinate analysis
