# Supplementary material for: Deciphering the roles of bacterial and fungal communities in the formation and quality of agarwood
Source: Stress Biol. 2024 Sep 20;4(1):40. doi: 10.1007/s44154-024-00179-5 (PMC11415328; doi:10.1007/s44154-024-00179-5)
Supplement: Supplementary file 1 — Supplementary Material 1: Figure S1. Analysis of the microbial community compositions and diversities in various A. sinensis tissues at the phylum level. Figure S2. Chord diagrams of the bacterial and fungal communities were generated for different tissue sites. Figure S3. Clustering analysis of the microbial species abundance at the class, order, family, and species levels in various A. sinensis tissues. [file 44154_2024_179_MOESM1_ESM.zip › 44154_2024_179_MOESM1_ESM.docx]

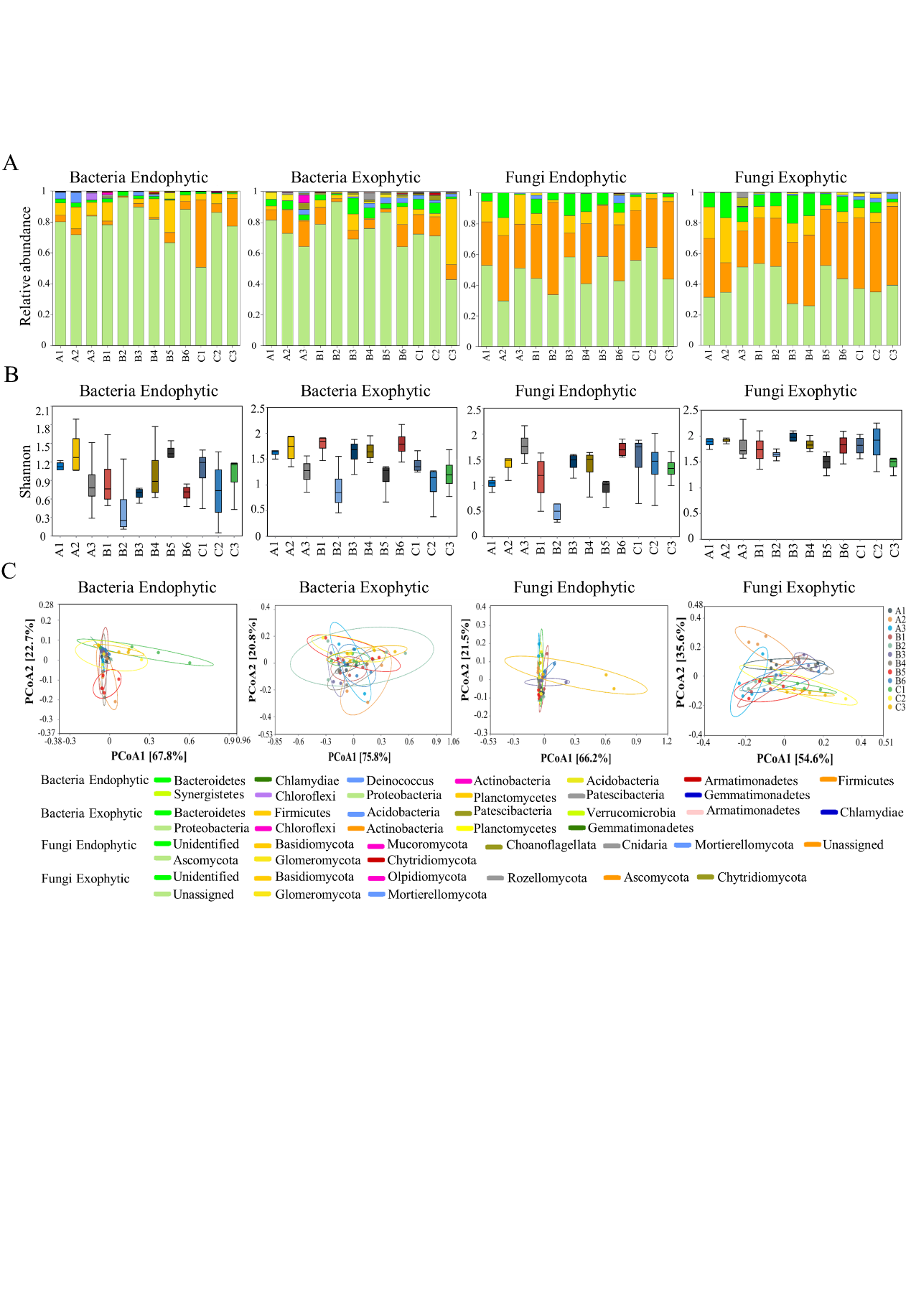


**Figure S1. Analysis of the microbial community compositions and diversities in various *A. sinensis* tissues at the phylum level.**

(A) The bacterial community compositions in different tissues. The horizontal axis represents the sample names, and the vertical axis represents the relative abundance. The stacked bars show the relative abundance at each taxonomic level, and the colours are used only to differentiate between different tissues.

(B) Analysis of the difference in the Shannon index among different plant tissues. The Shannon index is shown on the vertical axis, and sample names are shown on the horizontal axis. The median, dispersion, maximum, and minimum values of the species diversity within each group can be visually observed. The Kruskal‒Wallis rank-sum test (using the kruskal.test function in R) was used to evaluate the differences in the diversity indices between different groups.

(C) Principal coordinate analysis (PCoA) of the microbial communities in various tissues. Each point represents a sample, and points with the same colour come from the same group, while points with different colours represent different sample groups. The closer the distance between two points is, the smaller the difference in community composition between them, indicating that the community compositions of the sample groups are more similar. PCoA1 and PCoA2 are the two principal coordinate components, with PCoA1 representing the principal coordinate component that explains the largest possible amount of data variation and PCoA2 representing the principal coordinate component that explains the largest proportion of the remaining variance.

The first group consisted of 5-month-old *A. sinensis* seedlings (young leaf/A1, young branch/A2, young root/A3); the second group consisted of healthy 7-year-old *A. sinensis* (seed/B1, flower/B2, leaf/B3, branch/B4, bark/B5, trunk/B6); and the third group consisted of mature *A. sinensis* with agarwood (white trunk/C1, brown trunk/C2, agarwood bark/C3).


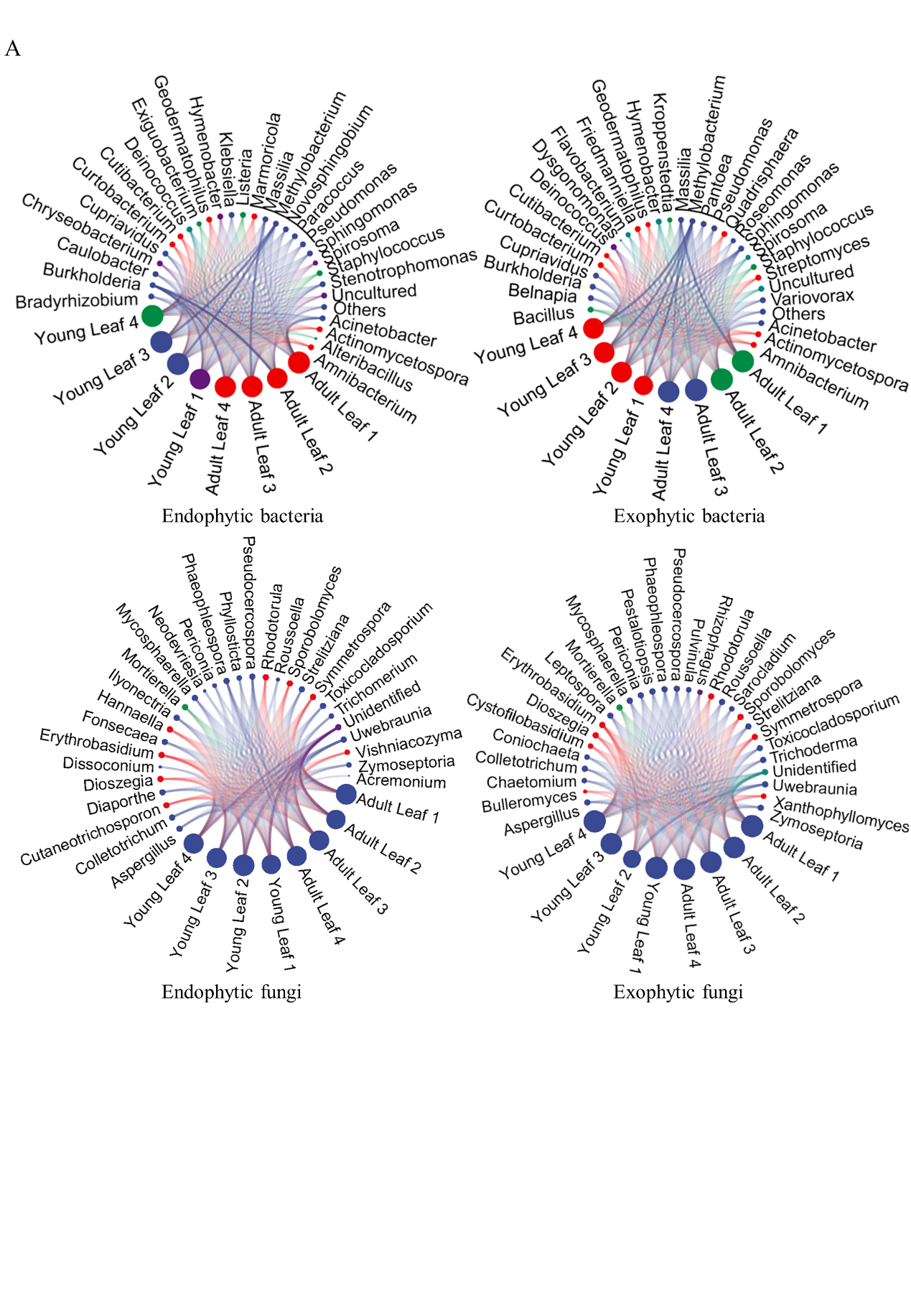


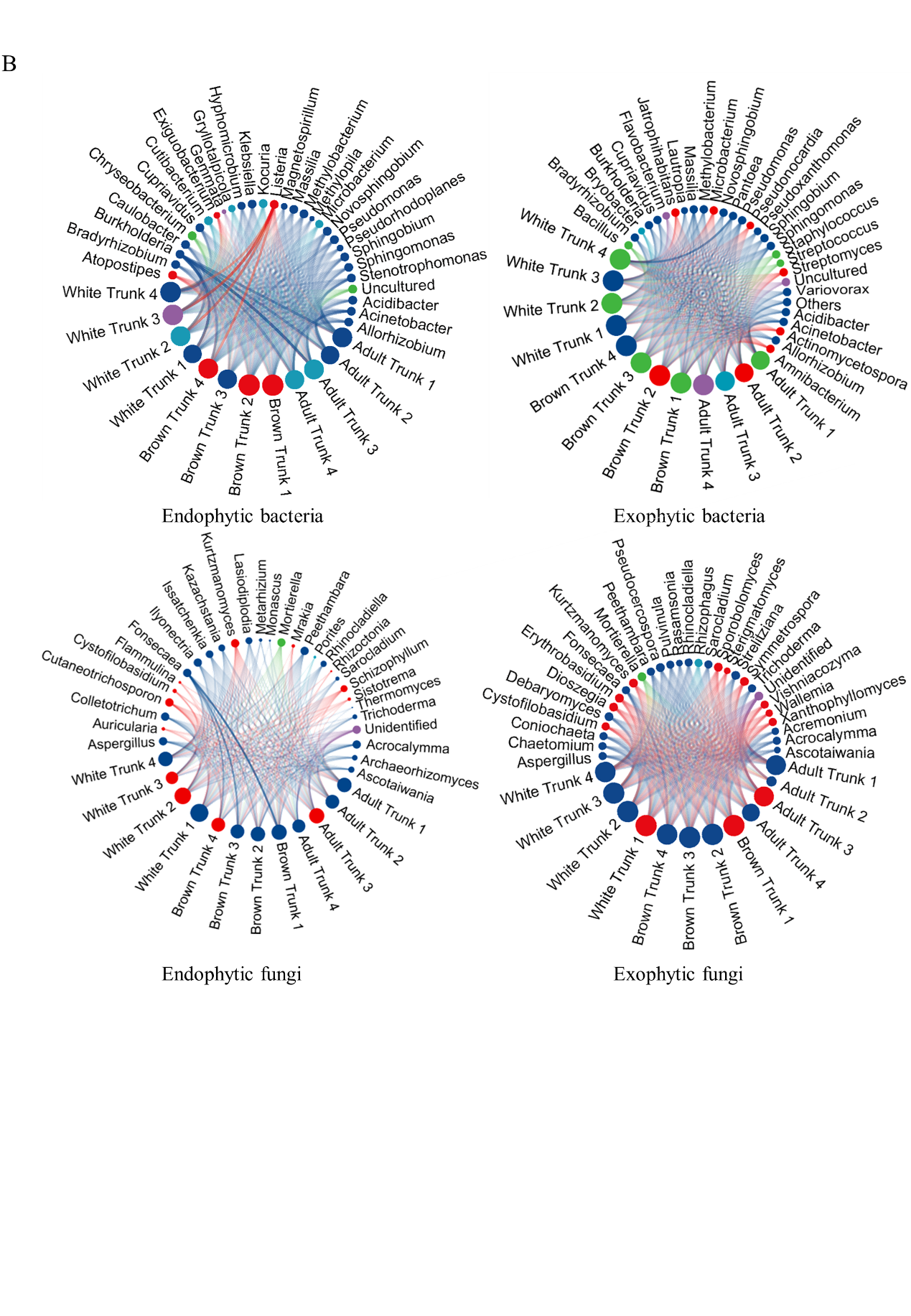


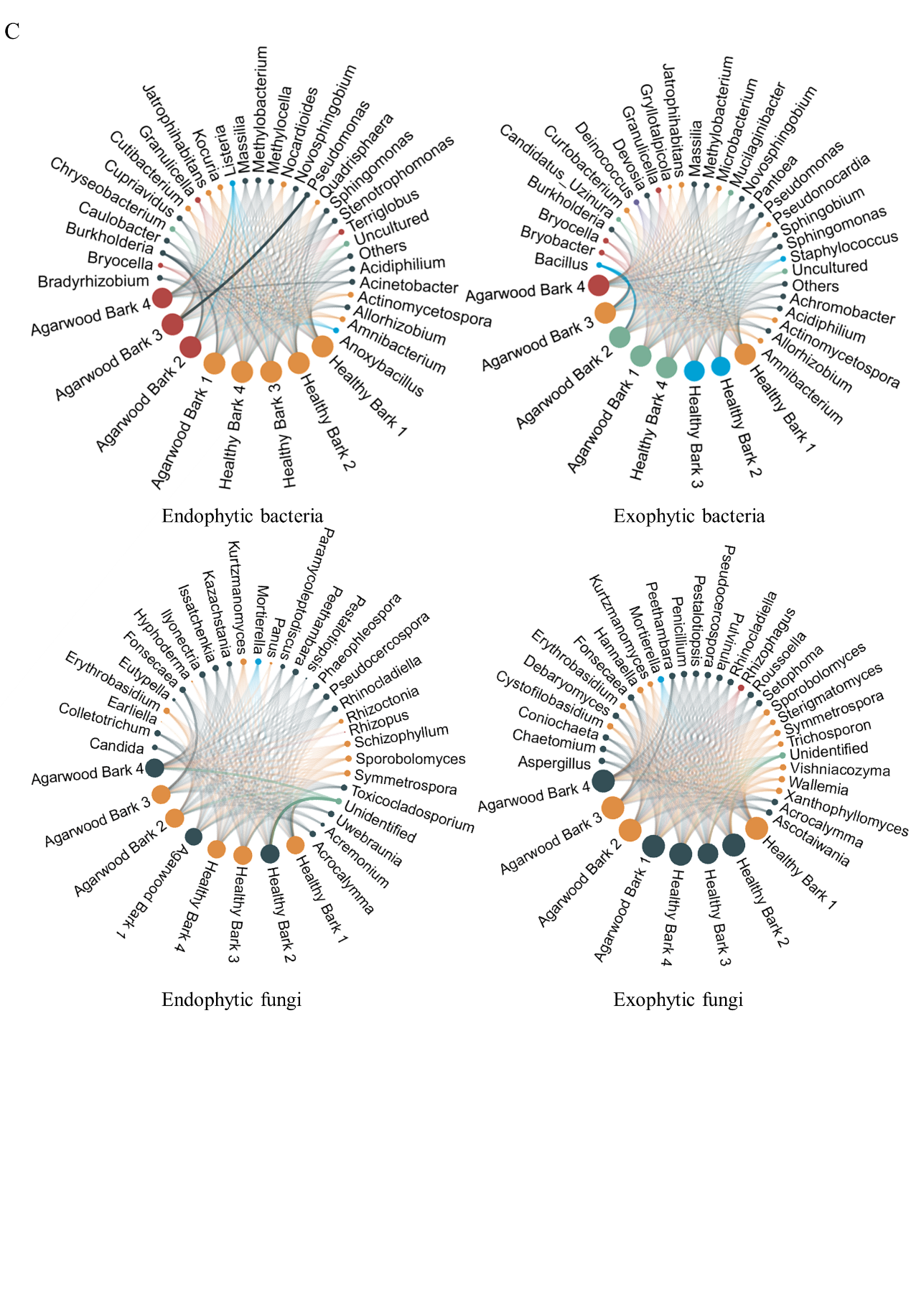


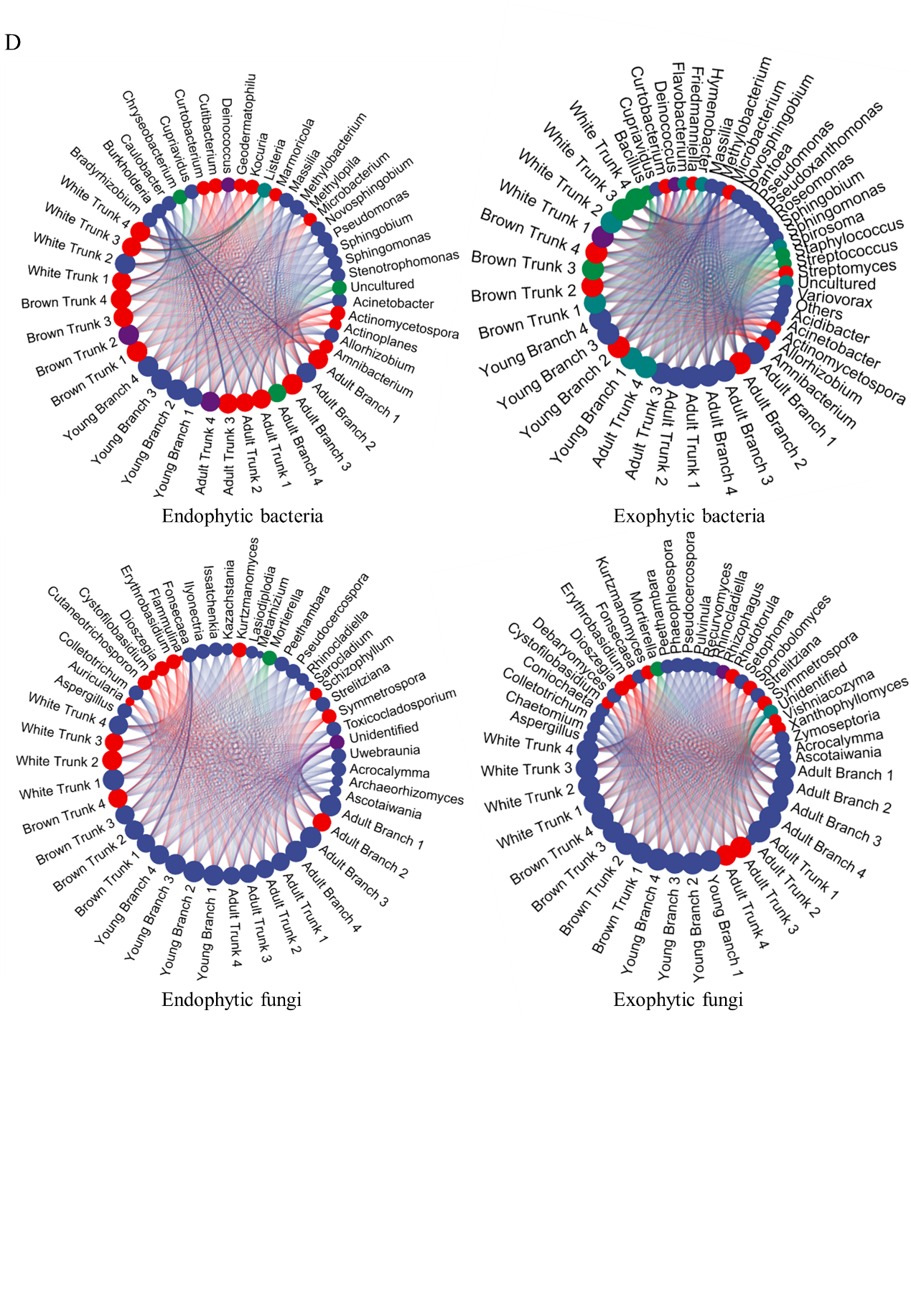


**Figure S2**. **Chord diagrams of the bacterial and fungal communities were analysed in different tissue sites.**

(A) Collinear chord diagram analysis of fungal/bacterial communities in young and adult leaves.

Four replicates of each tissue sample were used. The analysis encompassed four perspectives: endophytic bacteria, exophytic bacteria, endophytic fungi and exophytic fungi. There are two types of nodes in the chord diagram: species-nodes and sample-nodes. If a species appears in a sample, there is a line between the species and the sample. The color of the sample nodes follows the species nodes with the highest contribution. The size of all nodes is proportional to all their lines. The thickness and transparency of lines are related to abundance. The higher the relative abundance of the species in the sample, the thicker and more pronounced the lines between them, and vice versa.

(B) Collinear chord diagram analysis of fungal/bacterial communities in healthy and agarwood trunk.

Four replicates of each tissue sample were used. The analysis encompassed four perspectives: endophytic bacteria, exophytic bacteria, endophytic fungi and exophytic fungi. There are two types of nodes in the chord diagram: species-nodes and sample-nodes. If a species appears in a sample, there is a line between the species and the sample. The colours of the sample nodes follow the species nodes with the highest contributions. The sizes of all nodes are proportional to all their lines. The thickness and transparency of the lines are related to their abundance. The greater the relative abundance of the species in the sample is, the thicker and more pronounced the lines between them are, and vice versa.

(C) Collinear chord diagram analysis of the fungal/bacterial communities in healthy and agarwood bark.

Four replicates of each tissue sample were used. The analysis encompassed four perspectives: endophytic bacteria, exophytic bacteria, endophytic fungi and exophytic fungi. There are two types of nodes in the chord diagram: species-nodes and sample-nodes. If a species appears in a sample, there is a line between the species and the sample. The colours of the sample nodes follow the species nodes with the highest contributions. The sizes of all nodes are proportional to all their lines. The thickness and transparency of the lines are related to their abundance. The greater the relative abundance of the species in the sample is, the thicker and more pronounced the lines between them are, and vice versa.

(D) Collinear chord diagram analysis of fungal/bacterial communities in the branches and trunks.

Four replicates of each tissue sample were used. The analysis encompassed four perspectives: endophytic bacteria, exophytic bacteria, endophytic fungi and exophytic fungi. There are two types of nodes in the chord diagram: species-nodes and sample-nodes. If a species appears in a sample, there is a line between the species and the sample. The colours of the sample nodes follow the species nodes with the highest contributions. The sizes of all nodes are proportional to all their lines. The thickness and transparency of the lines are related to their abundance. The greater the relative abundance of the species in the sample is, the thicker and more pronounced the lines between them are, and vice versa.


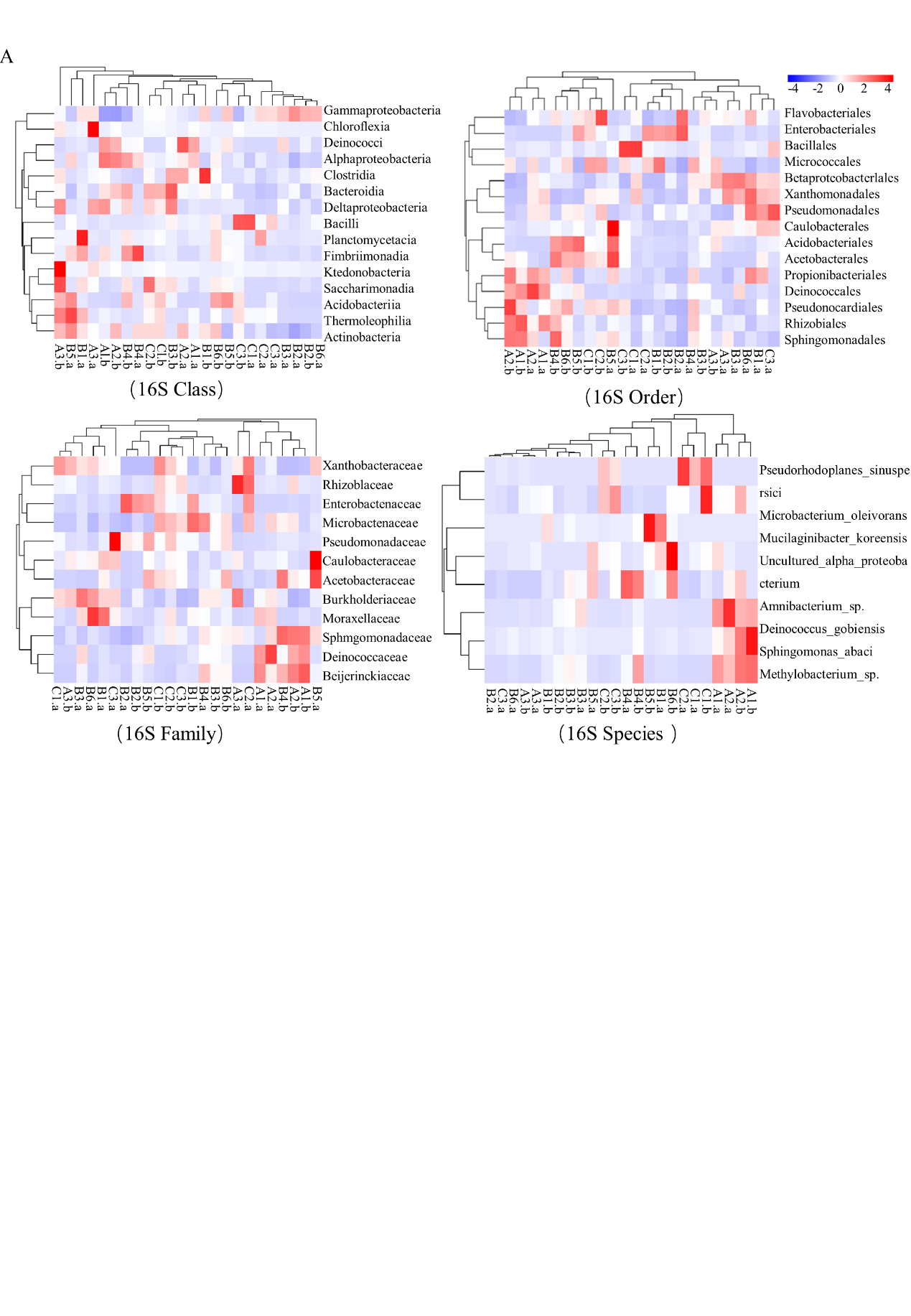


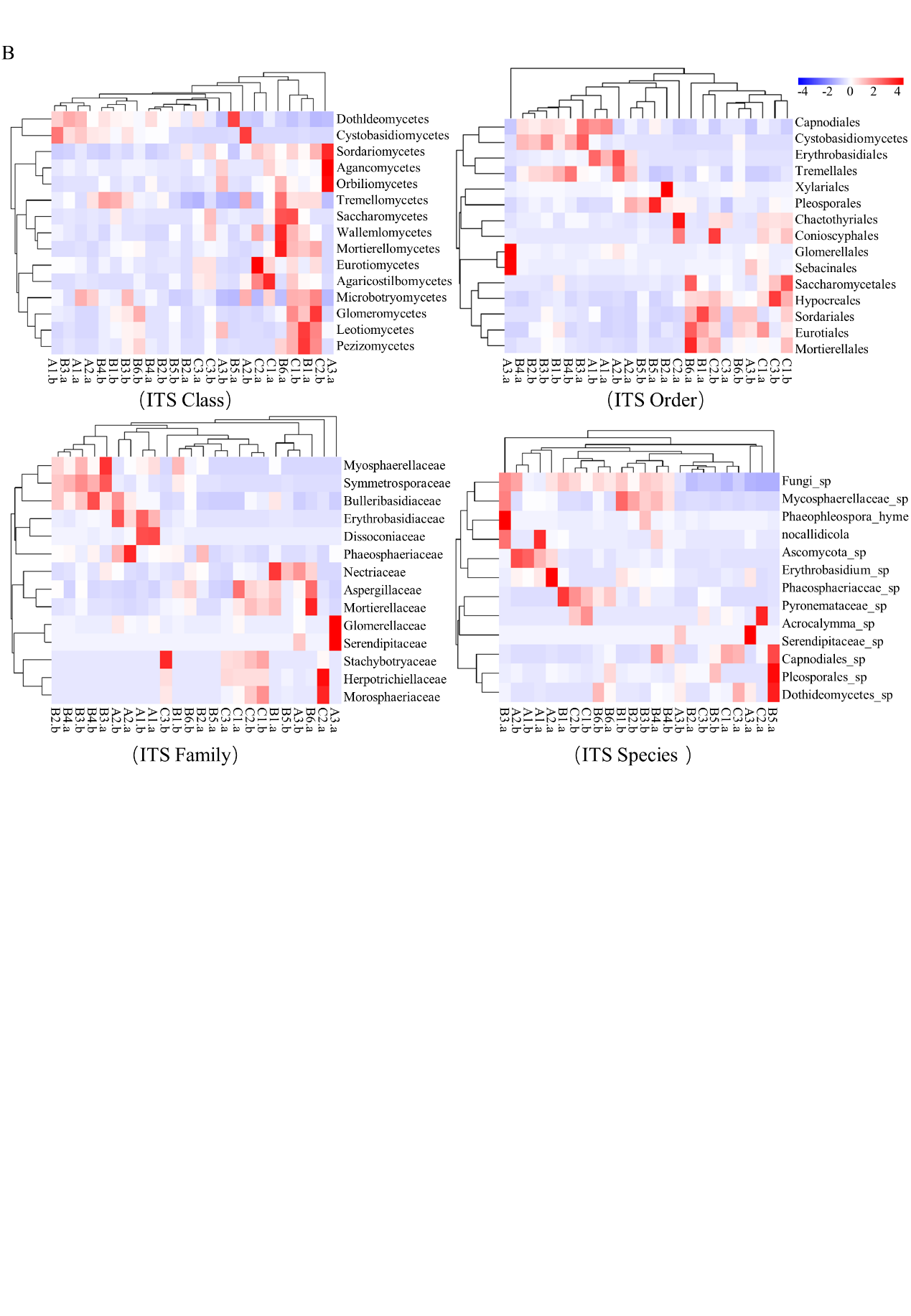


**Figure S3**. **Clustering analysis of the microbial species abundance at the class, order, family, and species levels in various *A. sinensis* tissues.**

(A) Cluster analysis of species abundance of endogenous and exogenous bacteria in different tissues across various taxonomic levels.

The horizontal axis represents the samples, and the vertical axis represents the species. The clustering tree on the left is the species clustering tree, and the clustering tree on the top reflects the similarity of the community composition between samples. The values corresponding to the middle squares are the standardized relative abundance of each row of species. The colour intensity of the squares represents the species abundances: the redder the square is, the greater the relative abundance of the species among the samples; the bluer the square is, the lower the relative abundance of the species among the samples. A horizontal comparison can be performed, but a vertical comparison cannot.

(B) Cluster analysis of species abundance of endogenous and exogenous fungi in different tissues across various taxonomic levels.

The horizontal axis represents the samples, and the vertical axis represents the species. The clustering tree on the left is the species clustering tree, and the clustering tree on the top reflects the similarity of the community composition between samples. The values corresponding to the middle squares are the standardized relative abundance of each row of species. The colour intensity of the squares represents the species abundances: the redder the square is, the greater the relative abundance of the species among the samples; the bluer the square is, the lower the relative abundance of the species among the samples. A horizontal comparison can be performed, but a vertical comparison cannot.

The first group consisted of 5-month-old A. sinensis seedlings (young leaf/A1, young branch/A2, young root/A3); the second group consisted of healthy 7-year-old A. sinensis (seed/B1, flower/B2, leaf/B3, branch/B4, bark/B5, trunk/B6); and the third group consisted of mature A. sinensis with agarwood (white trunk/C1, brown trunk/C2, agarwood bark/C3). “a” indicates an endophyte, and “b” indicates an exophyte.
