# Supplementary figures and images for: Deciphering the roles of bacterial and fungal communities in the formation and quality of agarwood
Source: Stress Biol. 2024 Sep 20;4(1):40. doi: 10.1007/s44154-024-00179-5 (PMC11415328; doi:10.1007/s44154-024-00179-5)

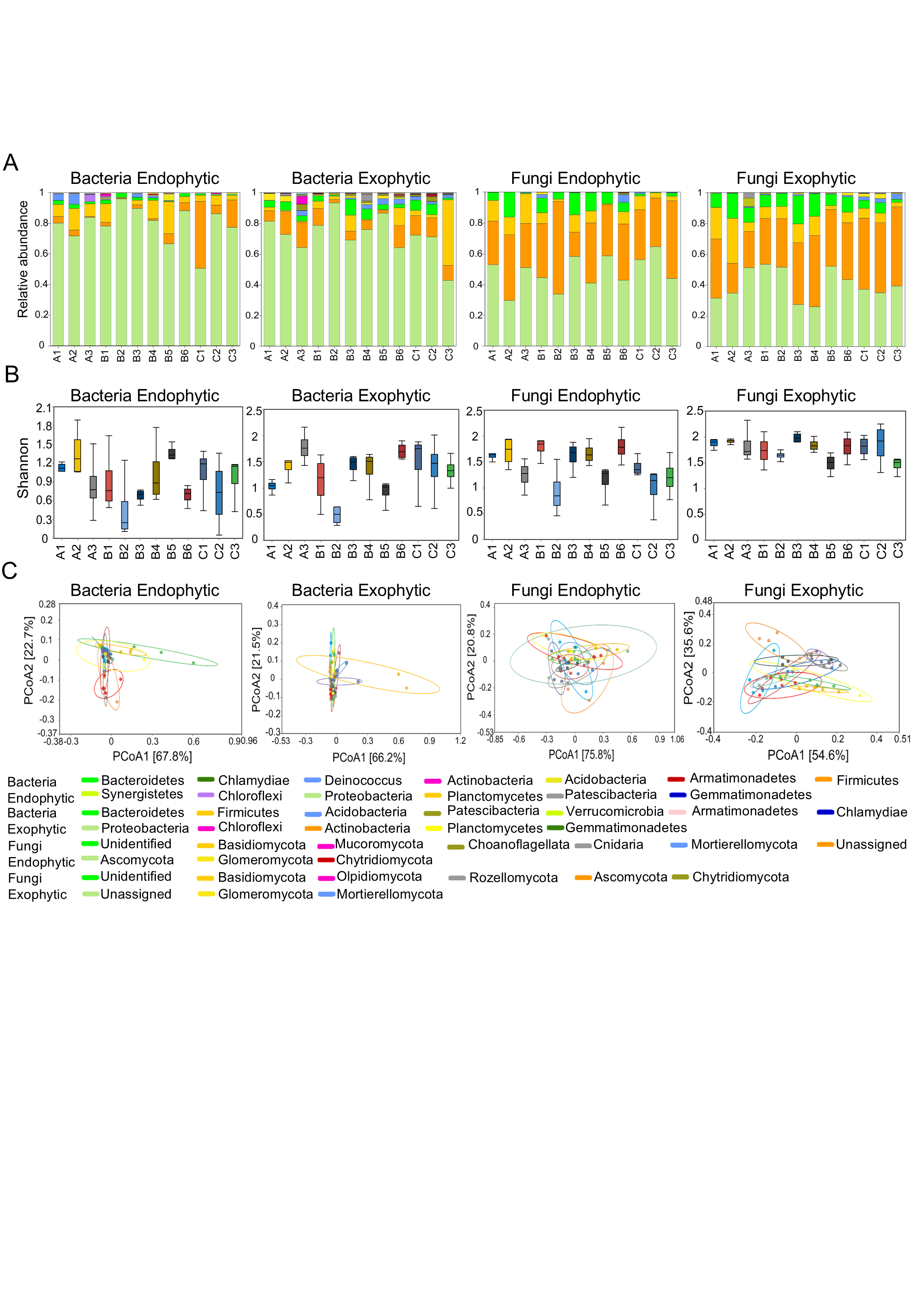

Supplement: Supplementary file 1 — Supplementary Material 1: Figure S1. Analysis of the microbial community compositions and diversities in various A. sinensis tissues at the phylum level. Figure S2. Chord diagrams of the bacterial and fungal communities were generated for different tissue sites. Figure S3. Clustering analysis of the microbial species abundance at the class, order, family, and species levels in various A. sinensis tissues. [file 44154_2024_179_MOESM1_ESM.zip › Supplementary-Figure S1_ESM.tif]

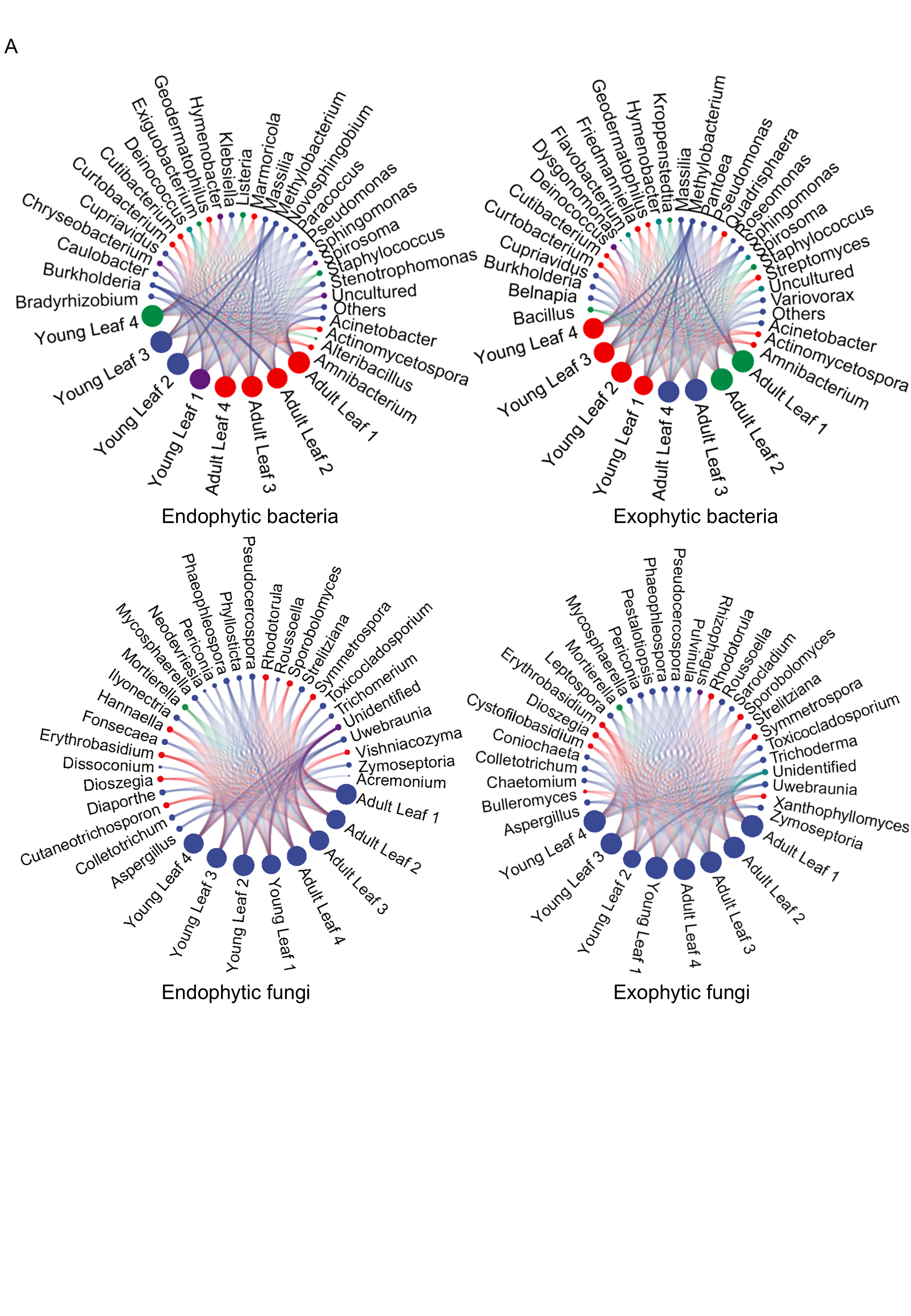

Supplement: Supplementary file 1 — Supplementary Material 1: Figure S1. Analysis of the microbial community compositions and diversities in various A. sinensis tissues at the phylum level. Figure S2. Chord diagrams of the bacterial and fungal communities were generated for different tissue sites. Figure S3. Clustering analysis of the microbial species abundance at the class, order, family, and species levels in various A. sinensis tissues. [file 44154_2024_179_MOESM1_ESM.zip › Supplementary-Figure S2-A_ESM.tif]

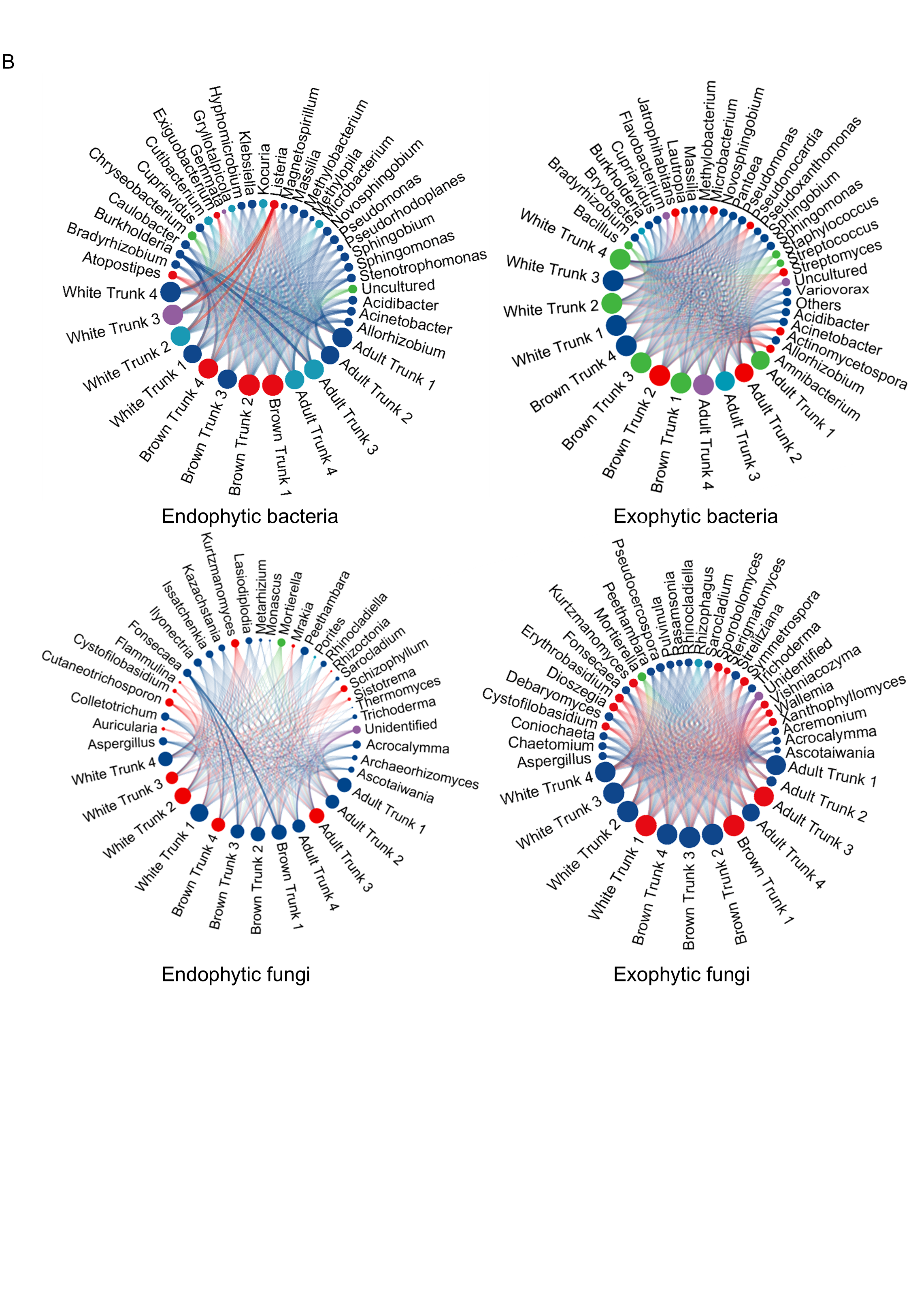

Supplement: Supplementary file 1 — Supplementary Material 1: Figure S1. Analysis of the microbial community compositions and diversities in various A. sinensis tissues at the phylum level. Figure S2. Chord diagrams of the bacterial and fungal communities were generated for different tissue sites. Figure S3. Clustering analysis of the microbial species abundance at the class, order, family, and species levels in various A. sinensis tissues. [file 44154_2024_179_MOESM1_ESM.zip › Supplementary-Figure S2-B_ESM.tif]

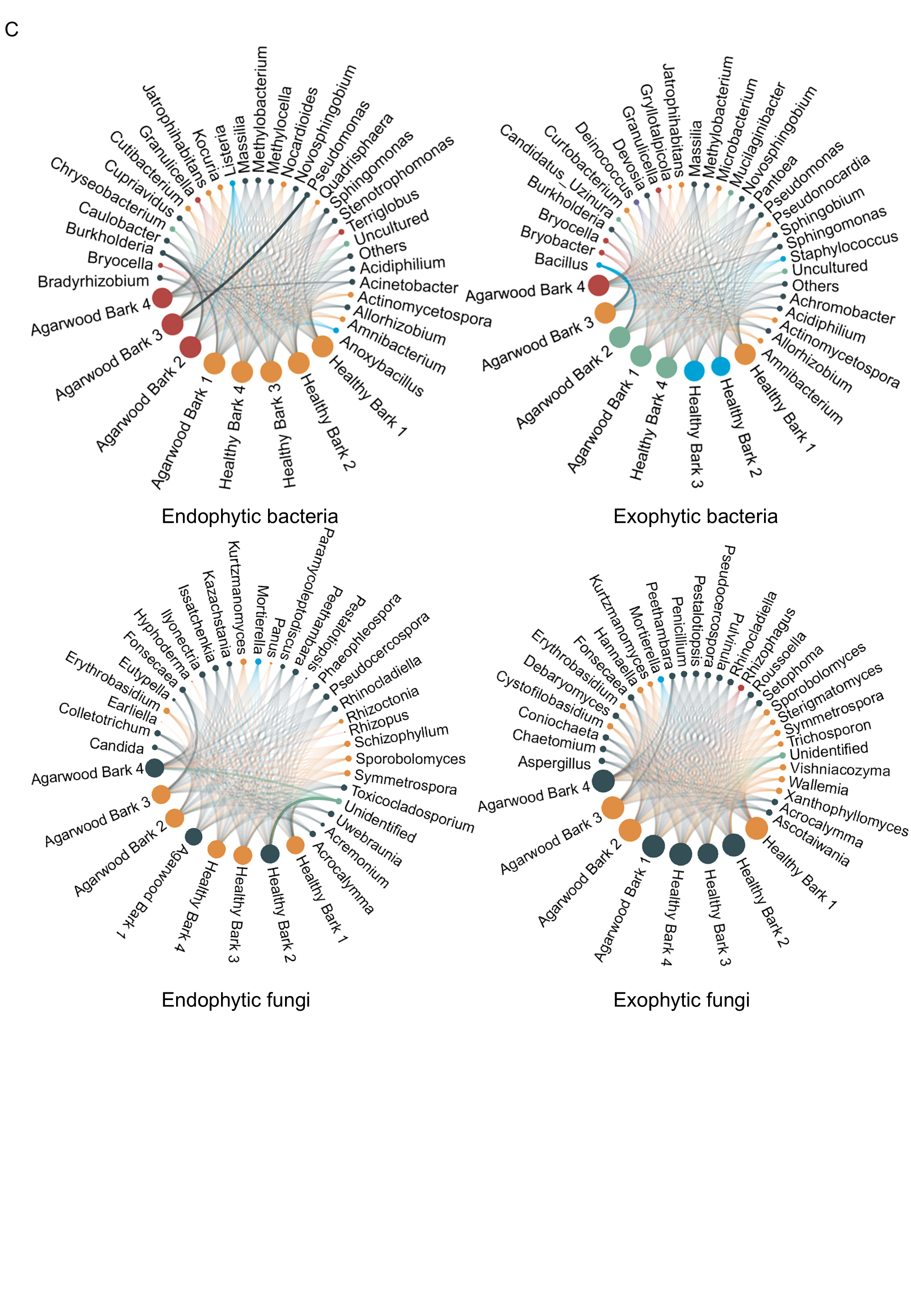

Supplement: Supplementary file 1 — Supplementary Material 1: Figure S1. Analysis of the microbial community compositions and diversities in various A. sinensis tissues at the phylum level. Figure S2. Chord diagrams of the bacterial and fungal communities were generated for different tissue sites. Figure S3. Clustering analysis of the microbial species abundance at the class, order, family, and species levels in various A. sinensis tissues. [file 44154_2024_179_MOESM1_ESM.zip › Supplementary-Figure S2-C_ESM.tif]

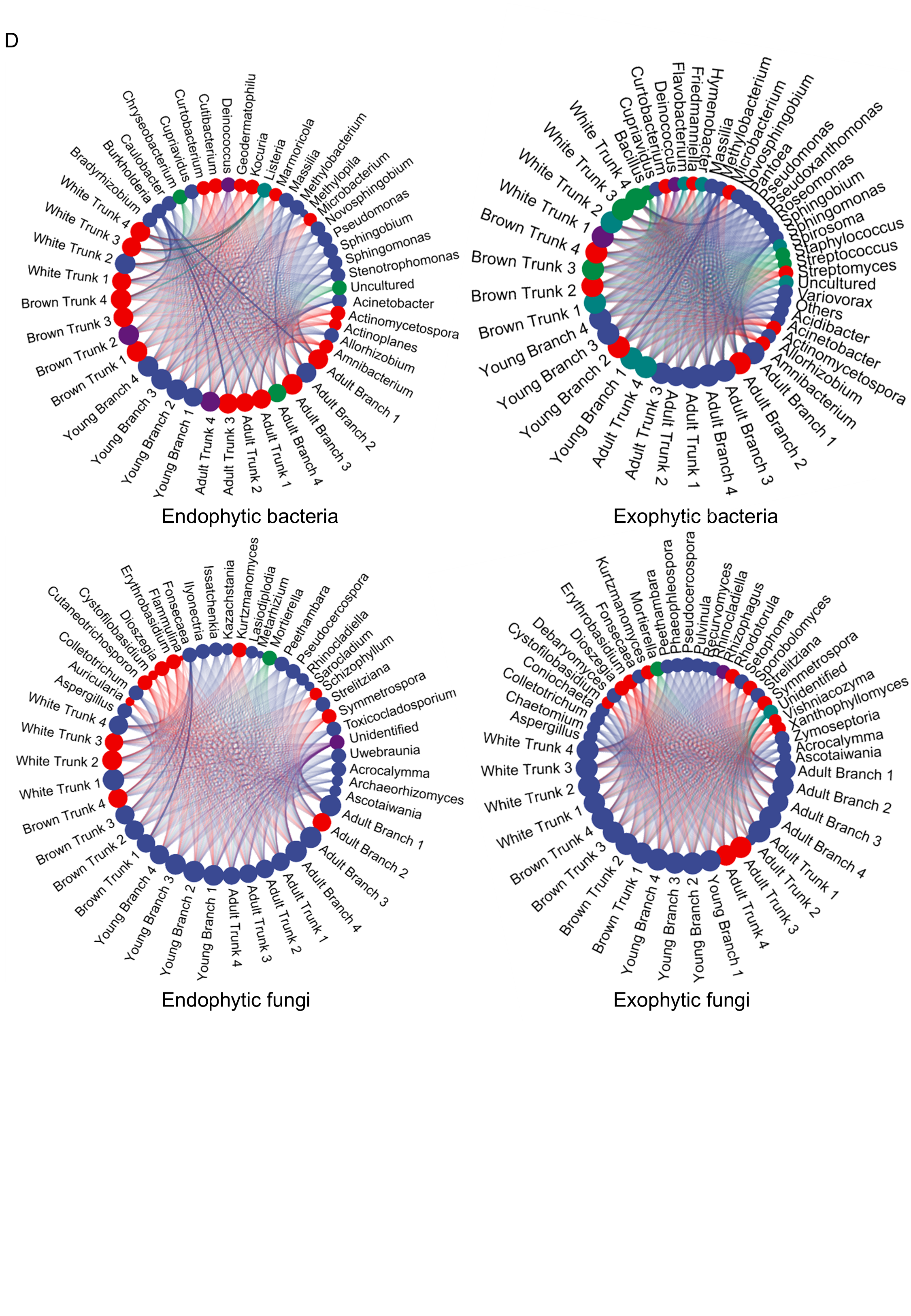

Supplement: Supplementary file 1 — Supplementary Material 1: Figure S1. Analysis of the microbial community compositions and diversities in various A. sinensis tissues at the phylum level. Figure S2. Chord diagrams of the bacterial and fungal communities were generated for different tissue sites. Figure S3. Clustering analysis of the microbial species abundance at the class, order, family, and species levels in various A. sinensis tissues. [file 44154_2024_179_MOESM1_ESM.zip › Supplementary-Figure S2-D_ESM.tif]

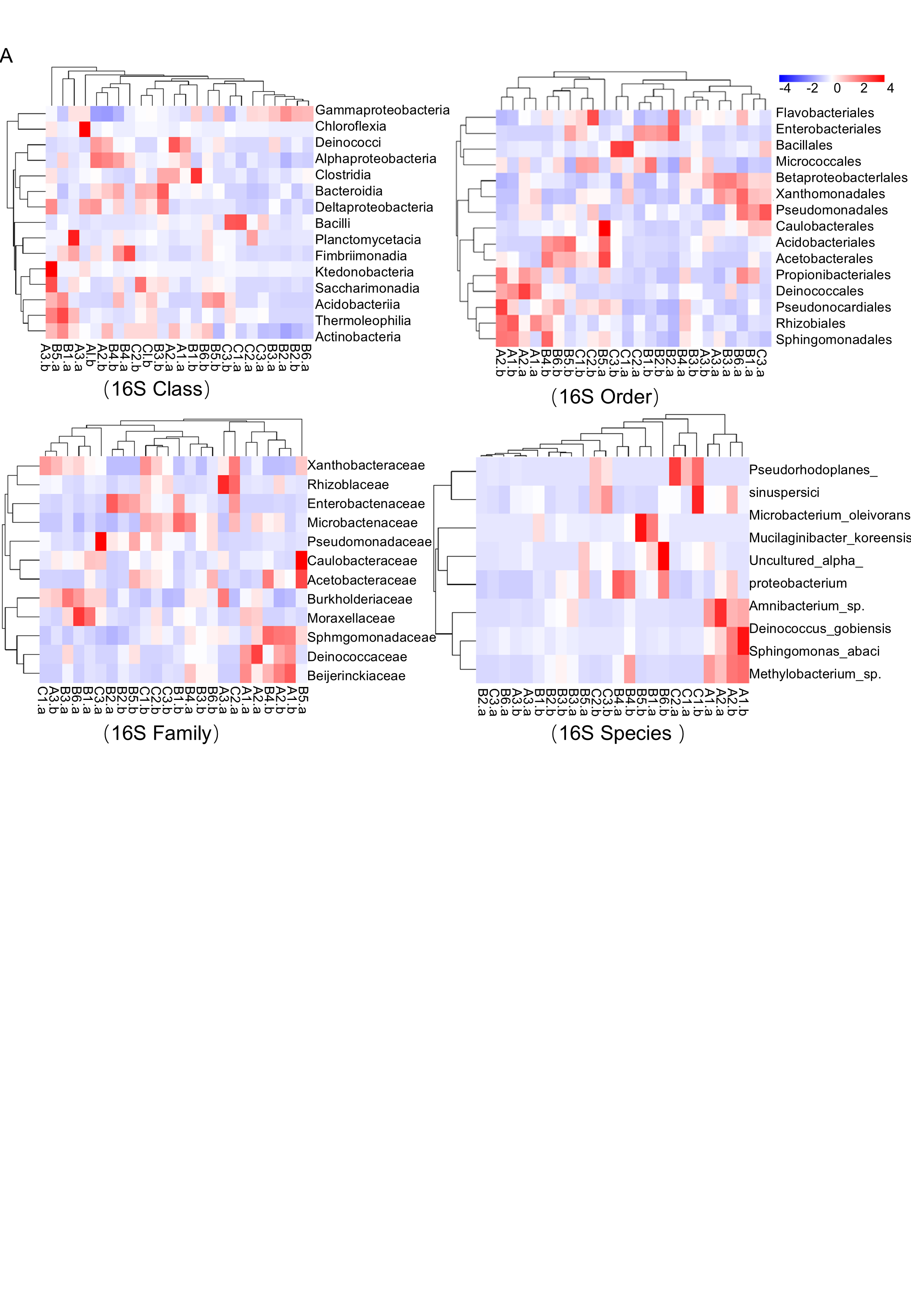

Supplement: Supplementary file 1 — Supplementary Material 1: Figure S1. Analysis of the microbial community compositions and diversities in various A. sinensis tissues at the phylum level. Figure S2. Chord diagrams of the bacterial and fungal communities were generated for different tissue sites. Figure S3. Clustering analysis of the microbial species abundance at the class, order, family, and species levels in various A. sinensis tissues. [file 44154_2024_179_MOESM1_ESM.zip › Supplementary-Figure S3-A_ESM.tif]

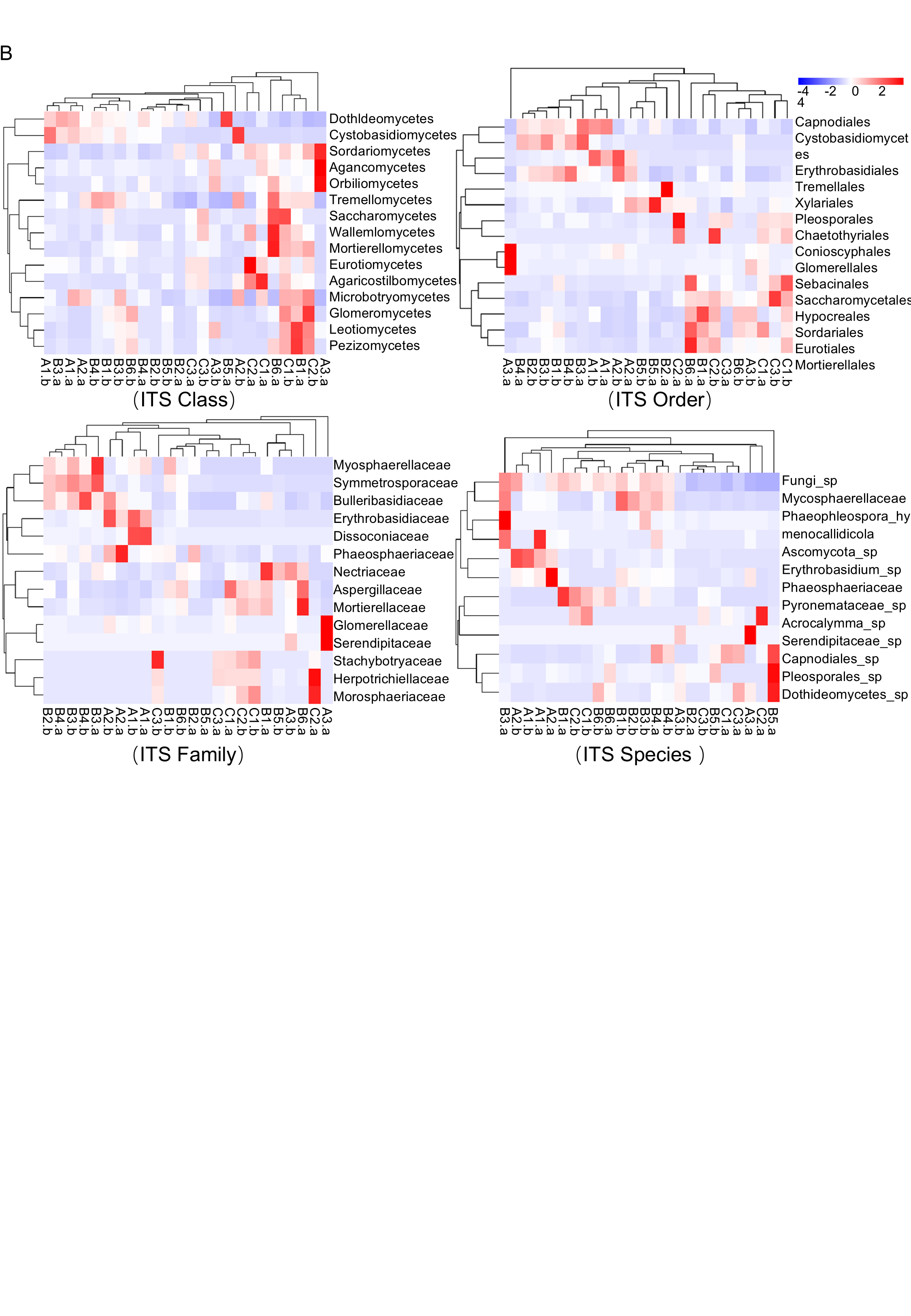

Supplement: Supplementary file 1 — Supplementary Material 1: Figure S1. Analysis of the microbial community compositions and diversities in various A. sinensis tissues at the phylum level. Figure S2. Chord diagrams of the bacterial and fungal communities were generated for different tissue sites. Figure S3. Clustering analysis of the microbial species abundance at the class, order, family, and species levels in various A. sinensis tissues. [file 44154_2024_179_MOESM1_ESM.zip › Supplementary-Figure S3-B_ESM.tif]
